# Large-Scale Characterization of the Soil Microbiome in Ancient Tea Plantations Using High-Throughput 16S rRNA and Internal Transcribed Spacer Amplicon Sequencing

**DOI:** 10.3389/fmicb.2021.745225

**Published:** 2021-10-15

**Authors:** Ling Kui, Guisheng Xiang, Ya Wang, Zijun Wang, Guorong Li, Dawei Li, Jing Yan, Shuang Ye, Chunping Wang, Ling Yang, Shiyu Zhang, Shuangyan Zhang, Ling Zhou, Heng Gui, Jianchu Xu, Wei Chen, Jun Zhang, Tingyuan Huang, Aasim Majeed, Jun Sheng, Yang Dong

**Affiliations:** ^1^Shenzhen Qianhai Shekou Free Trade Zone Hospital, Shenzhen, China; ^2^Yunnan Agricultural University Applied Genomics Technology Laboratory, School of Biological Big Data, Yunnan Agricultural University, Kunming, China; ^3^College of Food Science and Technology, Yunnan Agricultural University, Kunming, China; ^4^Lincang Tea Research Institute, Lincang, China; ^5^Longrun Pu’er Tea College of Yunnan Agricultural University, Kunming, China; ^6^CAS Key Laboratory for Plant Diversity and Biogeography of East Asia, Kunming Institute of Botany, Chinese Academy of Sciences, Kunming, China; ^7^Centre for Mountain Futures, Kunming Institute of Botany, Chinese Academy of Sciences, Kunming, China; ^8^College of Agronomy and Biotechnology, Yunnan Agricultural University, Kunming, China; ^9^Guangzhou Center for Disease Control and Prevention, Guangzhou, China; ^10^Molecular Genetics Laboratory, Central University of Punjab, Lahore, India; ^11^State Key Laboratory for Conservation and Utilization of Bio-Resources in Yunnan, Yunnan Agricultural University, Kunming, China; ^12^Yunnan Research Institute for Local Plateau Agriculture and Industry, Kunming, China; ^13^Key Laboratory for Agro-Biodiversity and Pest Control of Ministry of Education, Yunnan Agricultural University, Kunming, China

**Keywords:** *Camellia sinensis* var. *assamica*, microbiome, function, ancient tea plantations, 16S and ITS rRNA

## Abstract

There is a special interaction between the environment, soil microorganisms, and tea plants, which constitute the ecosystem of tea plantations. Influenced by environmental factors and human management, the changes in soil microbial community affected the growth, quality, and yield of tea plants. However, little is known about the composition and structure of soil bacterial and fungal communities in 100-year-old tea plantations and the mechanisms by which they are affected. In this regard, we characterized the microbiome of tea plantation soils by considering the bacterial and fungal communities in 448 soil samples from 101 ancient tea plantations in eight counties of Lincang city, which is one of the tea domestication centers in the world. 16S and Internal Transcribed Spacer (ITS) rRNA high-throughput amplicon sequencing techniques were applied in this study. The results showed that the abundance, diversity, and composition of the bacterial and fungal communities have different sensitivity with varying pH, altitude, and latitude. pH and altitude affect soil microbial communities, and bacterial communities are more sensitive than fungi in terms of abundance and diversity to pH. The highest α-diversity of bacterial communities is shown in the pH 4.50–5.00 and 2,200-m group, and fungi peaked in the pH 5.00–5.50 and 900-m group. Because of environmental and geographical factors, all microbes are similarly changing, and further correlations showed that the composition and structure of bacterial communities are more sensitive than fungal communities, which were affected by latitude and altitude. In conclusion, the interference of anthropogenic activities plays a more important role in governing fungal community selection than environmental or geographical factors, whereas for the bacterial community, it is more selective to environment adaptation than to adaptation to human activities.

## Introduction

*Camellia sinensis* L. is an evergreen shrub or small tree belonging to the family Theaceae whose leaves and leaf buds are used to produce tea. It is one of the important crops in the tropical and subtropical regions. It is a common source of tea all over the world and is cultivated widely across 49°N latitude to 33°S latitude (Sharma and Kumudini, 2018). China is the largest tea-producing country and has the largest area under tea cultivation in the world, accounting for 55.83 and 41.32% of the world total, respectively^1, 2^. Small-leaf tea and large-leaf tea (*C. sinensis* var. *assamica*) are the two main varieties of today’s cultivation^3^. The large-leaf tea cultivation has a long-standing history in China. The Bangwei transition type ancient tea tree that the Yunnan tea planting ancestors Pu’er people cultivated and domesticated represents the experienced cultivation strongly. As an important part of tea industry in China, Pu’er tea (leaf of *C. sinensis* var. *assamica*) is processed as raw materials from Yunnan’s large-leaved species of sun-dried green tea, which, as a unique tea in Yunnan province, contains lipid-lowering, antibacterial, and antiviral abilities (Zhang et al., 2012; Pedan et al., 2018).

The soil microbial community plays a dynamic role in the growth and vigor of cultivated plant species. The quality of the soil is determined not only by the physiochemical properties of the soil but also by its microbial profile. Thus, the diversity and structure of the microbial communities within the rhizosphere have a profound impact on the growth and yield of crops. In turn, the type and duration of the cultivated crop species would influence the microbial community. Therefore, soil and crop management are considered the most important factors affecting soil quality (Dang, 2002). The intensity and duration of tea planting have a significant impact on the microbial community structure, biomass, and its function, which is most likely achieved through soil acidification and fertilizer addition (Han et al., 2007). The nitrogen fertilizer application (NH3) significantly enhanced the tea yield but resulted in more soil acidification of the plantation sites (Li S. Y. et al., 2016; Yan et al., 2018; Yang et al., 2018). Tea thrives well in acidic soils, with an optimum pH range of 4.50–5.50. However, rapid acidification is becoming an issue, besides other factors (Yan et al., 2020). Recent evidence suggested that in comparison to the forests, the acidification of the cultivated tea plantations, receiving more fertilizers, has amplified during the past two to three decades. However, no significant change in acidification was experienced in the tea plantations receiving organic manure. Thus, only approximately 43.9% of the tea plantations in China have the optimal pH (4.50–5.50) (Yan et al., 2020). Acidification altered the chemical dynamics of the soil as the cation exchange capacity, the exchangeable Ca, K, and Mg content decreased. The exchangeable Al^3+^ content increased in comparison to the exchangeable H^+^, which resulted in increased Al extraction, thereby inhibiting tea plants from taking essential nutrients such as P, Fe, K, Ca, and Mg. Furthermore, the biogeochemical cycling of Al in tea leaf litter would accelerate further potential acidification of tea plantation soils. Besides inducing the loss of soil fertility, loss of number of important soil microorganisms, and loss of nitrogen and phosphorus content, the acidification of the tea plantation sites also resulted in the accumulation of organics in the roots and polyphenols plus heavy metals like Pb, Cu, and Cd in the leaves of tea plants. This would even become more intense with the increasing age of tea plants (Oh et al., 2006; Fung et al., 2008; Wang et al., 2010; Alekseeva et al., 2011; Li S. Y. et al., 2016; Li Y. C. et al., 2016; Yan et al., 2018; Yang et al., 2018). The prolonged monoculture of tea plantations leads to the gradual depletion of soil nutrient content, acidification, and deterioration of soil along with suppression of growth in tea plants (Wilson et al., 2004; Yan et al., 2018). This is also true for other annual crops like cucumbers (Zhou and Wu, 2012), potatoes (Liu et al., 2014), soybeans (Bai et al., 2015), and perennial crops such as apples (Mazzola and Manici, 2012), goldthread (Song et al., 2018), and coffee (Zhao et al., 2018). Taken together, these factors critically affected the growth, yield, and quality of the tea due to which the sustainable development of China’s tea industry has been constrained, besides economic losses to tea farmers. Therefore, this problem has attracted the attention of many soil ecologists and scientists (Zhao et al., 2012; Li Y. C. et al., 2016; Arafat et al., 2019; Tan et al., 2019).

Due to the large number of microorganisms in tea plantation soils, the complexity of their role, the variability of the soil itself, and the imperfection of research methods, relatively fewer studies are available on the microbial diversity of tea plantation soils. The development and refinement of molecular biology techniques, metagenomics, and high-throughput sequencing have greatly facilitated the study and understanding of the structural profile, diversity, and interactions of the microbiome. The microbiome within the rhizosphere of tea plants plays an important role in enhancing the soil quality, resilience of tea plants, and their resistance against pathogens (Zhao et al., 2012; Li Y. C. et al., 2016; Raaijmakers and Mazzola, 2016; Arafat et al., 2019; Tan et al., 2019). However, certain substances in the rhizosphere of tea plants inhibit microbial activity, with bacteria most sensitive than Actinomycetes and fungi (Pandey and Palni, 1996). In acidic tea plantation soils (pH < 6.0), the growth activity of soil microbes declines with decreasing pH (Koga et al., 2003), but it has also been found that tea plantation soil microbes are generally unaffected by soil pH, and certain research showed that 40- and 90-year-old tea tree soils contain only half the number of bacteria and fungi in contrast to 10-year-old tea tree soils, with bacteria predominating in the microbial community of the root soil (Wilson et al., 2004). Shannon diversity indices and richness were significantly lower in 8-, 50-, and 90-year-old tea plantations than in moorland, but both were significantly higher than in forest, and relatively high community diversity was found in 50-year-old tea plantations (Xue et al., 2006). The order of magnitude of the Shannon Diversity Index for soil microorganisms in tea plantations with four periods of different growth years was as follows: 45-year > 25-year > 7-year > 70-year tea plantations (Zhao et al., 2012). Generally, the application of compost affects the bacterial populations, and interestingly, when the compost is used in combination with nitrogen fertilizer, the soil microbial diversity increases (Qiu et al., 2014; Taha et al., 2016). As tea plants get older, change in composition and structure of the soil microbial communities occurs, and the species richness decreases, which in turn leads to a decrease in beneficial microorganisms along with an increase in soil pathogenic microorganisms (Li Y. C. et al., 2016; Arafat et al., 2019). The relative abundance of Ascomycota, Glomeromycota, and Chytridiomycota was reduced, whereas the relative abundance of Zygomycota and Basidiomycota was increased in the 2-, 15-, and 30-year-old monoculture history of replanted tea plantations. Furthermore, the beneficial fungi like *Mortierella alpina* and *M. elongatula* were gradually reduced along with an increase in pathogenic forms such as *Fusarium oxysporum*, *F. solani*, and *Microidium phyllanthi* in the soil of continuously grown tea plantations (Arafat et al., 2019). The bacterial genetic diversity index of tea plantation soils was lower than that of wasteland soils. The soils of organic tea plantations had higher ecosystem versatility, the soil microbial richness was significantly higher than that of unpolluted tea plantations and conventional tea plantations, and there were significant differences in soil bacterial community structure between the three tea plantations. The analysis of 16S rRNA gene sequences revealed that the dominant bacterial groups in tea plantation soils were Gamma and Alpha and Acidobacteria. The more alike the environmental variables are, the more similar the bacterial community structure in tea plantation soils is. It was found that soil organic carbon (SOC), nitrous nitrogen (NO3-N), and pH were the key soil factors affecting the bacterial community structure in tea plantation soils (Zhao et al., 2012; Qiu et al., 2014; Li Y. C. et al., 2016; Tan et al., 2019).

Lincang city is named for its proximity to the Lancang River, located in the southwestern part of Yunnan province, belonging to the subtropical low-latitude plateau mountain monsoon climate. It is the main stop on the ancient Tea-Horse Road, as well as one of the largest and most representative areas of the ancient tea heritage stock. The vast majority of the current research on tea soil microbes remained focused only on bacterial diversity (16S) and less on fungal diversity (ITS), and there has been less research on altitude drivers. Also, to the best of our knowledge, there are no data available for large samples and cross-regional studies of microbial populations in tea tree soils. With the popularity of high-pass sequencing, 16S and ITS amplicon sequencing have become important tools for studying the composition of microbial communities in environmental samples. In this study, we investigated the soil bacterial and fungal community dynamics of 101 representative ancient tea plantation sites in eight districts and counties of Lincang city. This study aimed to determine the effects of different gradients of pH, altitudes, and geographic locations on the microbial diversity, structure, and taxonomic composition of the tea plantations, and to explore the possible environmental factors that contribute to changes in soil microbial communities. Moreover, microbial classification was used to determine the presence of core microbiomes in tea plantations at different places along with the determination of their relative abundance and their potential role as hub taxa.

## Materials and Methods

### Sample Collection

**FIGURE 1:**
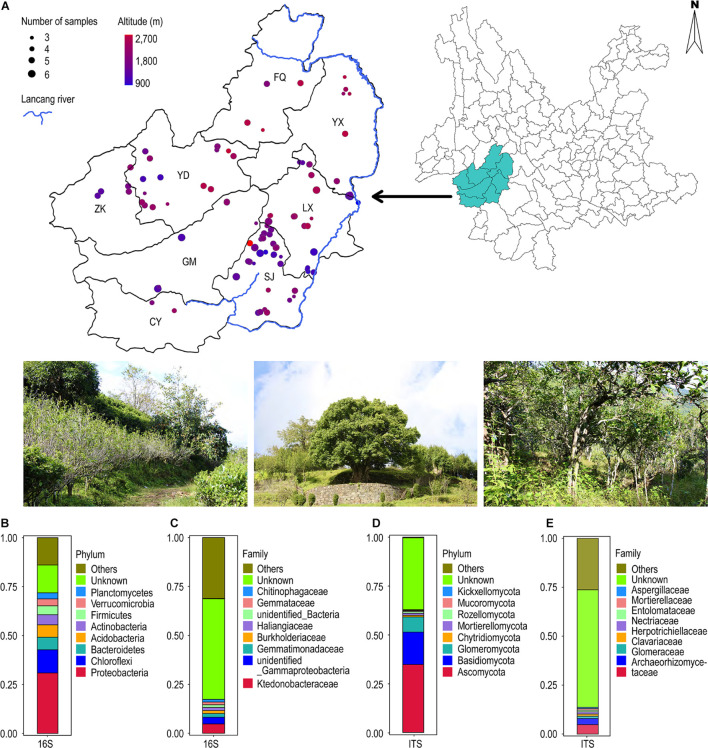
**(A)** Geographical distribution of 101 ancient tea plantations in Lincang city. **(B–E)** Annotation of all soil sample OTUs at the bacterial and fungal phylum and family classification level. **(B)** 16S (first 10 bacterial phylum); **(C)** 16S (first 10 bacterial family); **(D)** ITS (first 10 bacterial phylum); **(E)** ITS (first 10 bacterial family).

A total of 448 soil samples from the rhizobiome of 101 ancient tea plantation sites were collected from eight different counties and districts of Lincang city, Yunnan province in October 2019. The geographic coordinates (latitude and longitude) and elevation of the sampling sites were determined through GPS (JIEWEISEN, WS-009). We used real drone visualizations and map surveys for designing an appropriate sampling strategy. The details of the sampling locations are presented in Figure 1A. The soil was taken at a depth of approximately 20 cm from the surface. Stones and other impurities were removed. Each sample was collected in triplicate; two in 2-ml sterile tubes and the third in a 50-ml sterile centrifuge tube. The collected samples were snap-frozen in liquid nitrogen and carried to the lab.

### Determination of Soil pH

The collected soil samples were air-dried and sieved through a 0.20-mm nylon sieve. The soil and water were then mixed in the ratio of 1:2.5 [4 g of soil and 10 ml of ultrapure water (w/v)] in a 25-ml centrifuge tube. The mixture was vortexed at a maximum frequency for 10 min, left at room temperature for 30 min, followed by determination of pH through a pH meter (METTLER TOLEDO, FiveEasy Plus) using glass electrodes. The reading for each sample was taken five times, and the average was taken as the pH value of the samples (Arafat et al., 2019; Yan et al., 2020).

### DNA Extraction, Library Preparation, and Sequencing

Total DNA was extracted from each sample by following the instructions of the Omega Biotek EZNA^®^ Soil DNA Kit. The quality and quantity of the isolated DNA were determined through agarose gel electrophoresis and Nanodrop Photospectrometer, respectively (Thermo Fisher, Nanodrop 2000). A final working concentration of 1 ng/μl was used for downstream analysis. We used the primers F341 (5′-CCTAYGGGRBGCASCAG-3′) and R806 (5′-GGACTACNNGGGTATCTAAT-3′) (Stephen et al., 2017) for amplifying the V3–V4 region of the 16S rRNA gene. Moreover, the primers ITS-1F (5′-CTTGGTCATTTAGAGGAAGTAA-3′) and ITS-1R (5′-GCTGCGTTCTTCATCGATGC-3′) (Gardes and Bruns, 1993) were used to amplify the ITS1 region. All the primers were barcoded. Amplification of the 16S rRNA and ITS1 regions were performed through PCR reactions using Phusion^®^ High-Fidelity PCR Master Mix, New England Biolabs. Each PCR reaction consisted of Phusion Master Mix (15.0 μl), primers (1.5 μl), genomic DNA (10.0 μl), and dd H2O (2.0 μl). The thermocycler program consisted of an initial denaturation temperature of 98°C for 1 min, followed by 30 cycles each with 98°C for 10 s, 50°C for the 30 s, and 72°C for 30 s, followed by a final 72°C for 5 min. The PCR products were purified with Qiagen Gel Extraction Kit (Qiagen, Germany).

Then, the sequencing library was generated using TruSeq^®^ DNA PCR-Free Sample Preparation Kit (Illumina, United States) following the manufacturer’s recommendations. The index codes were added to the library. A total of 448 libraries were generated. The quality of the libraries was assessed through the Qubit@ 2.0 Fluorimeter (Thermo Fisher Scientific, United States) and Agilent Bioanalyzer 2100 system (Agilent Technologies, United States). Subsequently, each library was sequenced on the Illumina NovaSeq 6000 platform and 250-bp paired-end reads were generated.

### Sequence Data Processing

The paired-end raw reads were grouped based on their barcode sequences. Then, the paired reads were merged to obtain amplicon sequences using FLASH (version 1.2.7) (Magoč and Salzberg, 2011). The barcode and primer sequences as well as the low-quality amplicon sequences were removed using QIIME2 (v2019.10) (Bolyen et al., 2019). We used UCHIME (Quast et al., 2013) (parameter default) to detect and remove the chimeric sequences. Thus, clean amplicon data were generated, which were used for downstream analysis. The quality amplicons were then clustered into OTUs using Uparse (v10.0.240) (Edgar, 2013), based on a similarity threshold value of ≥97%. A representative sequence of each OTU was selected for further analysis. The representative sequences were aligned against the SILVA 132 database (for 16S rRNA sequences) and the UNITE database (for ITS sequences) (Abarenkov et al., 2010) to determine their taxonomic affiliations. Furthermore, to investigate the phylogenetic relationships among the OTUs, multiple sequence alignments were performed using Muscle (v3.8.31) (Edgar, 2004). QIIME2 (v2019.10) was used to calculate Weighted Unifrac distances and to construct UPGMA clustering trees.

### Statistical Analysis and Data Visualization

α-diversity represents the diversity within a sample, including species richness and species evenness measurements. We used QIIME2 (v2019.10) to calculate the sample Chao1 and Shannon indices to evaluate the complexity and diversity of species within the sample. The statistical evaluation of differences in α-diversity between multiple groups was carried out through the Kruskal–Wallis test with a *p*-adjusted cutoff value of 0.05, using agricolae package. β-diversity represents the differences in the microbiome among the samples. We employed non-metric multidimensional scaling (NMDS) test, Anosim, and MRPP analysis using the R vegan package (Philip, 2003; R Core Team, 2020) to calculate β-diversity measurements. We used correlation analysis to identify associations between taxa and environmental factors, such as pH, longitude and latitude, and altitude, using the corr.test function of the psych R package. The correlations were visualized using the pheatmap R package. Moreover, a redundancy analysis (RDA) and Mantel test were performed using the R vegan package to compare the correlations of environmental factors with microbial community composition and structure.

## Results

### Geographic, Elevation, and pH Range of the Samples

Analysis of geographic locations of the 448 samples from 101 tea plantation sites revealed that they were distributed between 23–25°N latitude and 98–101°E longitude. Their elevation ranged from 900 to 2,700 m (Figure 1A). The pH values ranged from 3.90 to 6.30, with an average value of 4.68. We created three pH gradients; pH < 4.50, pH between 4.50 and 5.50, and pH > 5.50. Our analysis revealed 39 sites with pH < 4.50, 58 sites with pH between 4.50 and 5.50, and 4 sites with pH > 5.50 (Supplementary Figure S1).

### Composition and Structure of Bacterial and Fungal Communities

After merging of paired-end raw reads, their quality control analysis, and filtration of chimeric sequences, a total of 84,691 active amplicon sequences were obtained belonging to the 16S rRNA region. Similarly, a total of 53,228 active sequences were obtained for the ITS region from 448 soil samples. The sequences were clustered to 21,293 OTUs of bacteria belonging to 65 phyla, and 14,978 OTUs of fungi belonging to 17 phyla, at a threshold of 0.97. The sparse curve of the OTUs gradually flattened out (Supplementary Figure S2), which indicated that the sequencing depth covered all the species in the sample.

The taxonomic annotation of OTUs was performed from phylum to species level (Figure 1). In case of bacteria, Proteobacteria (30.9%) was the most dominant phylum followed by Chloroflexi (11.9%), Bacteroidetes (6.4%), Acidobacteria (6.3%), Actinobacteria (5.2%), and Firmicutes (4.6%). Approximately 14.2% OTUs remained unclassified (Figure 1B). Ktedonobacteraceae (4.7%) was the dominant family followed by unidentified Gammaproteobacteria (3.5%) and Gemmatimonadaceae (1.9%). Approximately 53.1% OTUs could not be assigned to any family (Figure 1C). Similarly, in the case of fungi, Ascomycota (34.7%) was the most frequent phylum followed by Basidiomycota (16.6%) and Glomeromycota (7.7%). The unclassified OTUs accounted for up to 36.9% (Figure 1D). Archaeorhizomycetaceae (4.8%) was the dominant family, followed by Glomeraceae (3.2%) and Clavariaceae (1.2%), with 60.0% remaining unclassified (Figure 1E).

### Community Structure and Diversity in Response to Variations in pH

**FIGURE 2:**
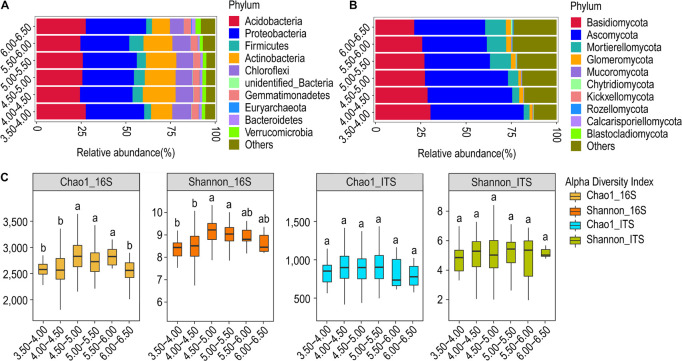
Relative abundance and α-diversity indices of soil bacterial and fungal communities in different pH gradient groups. **(A)** Relative abundance of bacteria at the phylum classification level (first 10 bacterial phylum, same below); **(B)** relative abundance of fungi at the phylum classification level (first 10 fungi phylum, same below); **(C)** Chao1 and Shannon indices of bacteria and fungi. Different lowercase letters indicate significant differences between subgroups (*p* < 0.05) (same below).

We estimated the abundance of bacteria and fungi and analyzed its relationship with variations in pH. Six pH gradients were used during the analysis. We observed that there is no significant trend (ascending or descending) in the relative abundance of bacterial phyla with pH (Figure 2A). Acidobacteria, Actinobacteria, and Proteobacteria have relative abundance > 10% at all pH gradients. Actinobacteria and Acidobacteria showed an opposite trend at the lower (pH 3.50–4.00) and upper (pH 6.00–6.50) pH extremes, with the former exhibiting less relative abundance and the latter showing greater relative abundance. However, Spearman’s correlation analysis did not show any significant relationship of the relative abundance of the bacterial phyla with variations in pH.

The fungal phyla, however, showed a clear relationship of the relative abundance with pH (Figure 2B). The relative abundance of Ascomycota and Basidiomycota showed a progressive decline with increasing pH. The trend is more explicit and contrasting in the case of Mortierellomycota, whose abundance increases toward basic pH. At the genus level, the relative abundance and composition of genera in bacteria and fungi were similar in all groups except for the highest pH group. *Arthrobacter* and *Archaeorhizomyces* were the dominant bacterial and fungal genera in all groups, respectively (Supplementary Figure S3A). The relative abundance of the genus *Hygrocybe* was substantial in all groups, except the group with pH > 6.0. *Penicillium* and *Russula* were important fungal genera in the low pH group (pH 3.5–4.0) whereas the genus *Mortierella* is important in the high pH group (pH > 6.0) (Supplementary Figure S3B).

The α-diversity analysis revealed that both the Chao1 and Shannon’s index of the bacterial community were significantly higher for the pH = 4.50–5.00 and 5.00–5.50 than for the pH = 3.50–4.00 and 4.00–4.50 (*p* < 0.05). In contrast, the Chao1 and Shannon’s index of the fungal community did not differ between the subgroups (Figure 2C). The number of OTUs was found to be highest for both the bacterial and fungal communities at pH = 4.50–5.00. The number of OTUs common to all the groups was observed to be 6,073 and 2,260 for bacterial and fungal communities, respectively (Supplementary Figure S4). The β-diversity analysis of microbial communities at different pH gradients through NMDS could not establish any distinctions in both bacterial and fungal communities (Supplementary Figure S5). The Anosim and MRPP test also did not reveal any clear trend in between-group or within-group differences. However, the test was statistically significant for some comparisons, although the correlations were weak (Supplementary Table S1).

UPGMA clustering revealed that the pH gradients of 4.50–5.00 and 5.00–5.50 formed one sub-cluster, so their community profile tends to be highly similar. Other pH gradients segregated into individual leaves. The gradient 6.00–6.50 was found most distinct from others, followed by the gradient 3.50–4.00, suggesting their distinct community structure as compared to other intermediary gradients (Supplementary Figure S6A). The results were slightly different in the case of fungi, with an upper gradient (pH = 6.00–6.50) quite distinct from others. Furthermore, it was revealed that the pH gradients of 5.00–5.50 and 5.5–6.00 host similar fungi, whereas the pH gradients of 3.50–4.00, 4.00–4.50, and 4.50–5.00 have their fungal profile (Supplementary Figure S6B).

### Community Structure and Diversity in Response to Variations in Elevation

**FIGURE 3:**
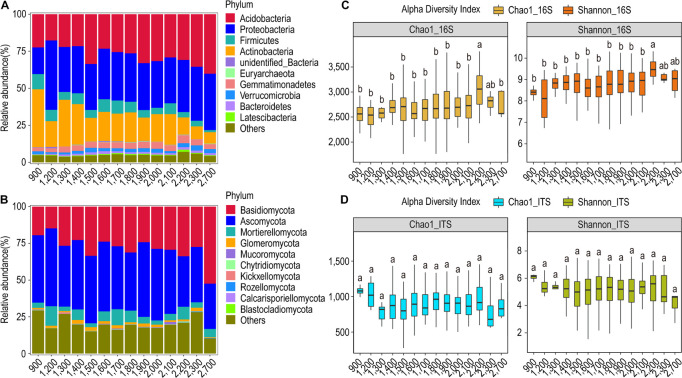
Relative abundance and α-diversity indices of soil bacterial and fungal communities in different altitude groups. **(A)** Relative abundance of bacteria at the phylum classification level; **(B)** relative abundance of fungi at the phylum classification level; **(C)** Chao1 and Shannon indices of bacteria and fungi. **(D)** Different lowercase letters indicate significant differences between subgroups (*p* < 0.05).

We observed that the overall structural profile of the bacterial and the fungal communities at 14 different altitudinal zones is similar, and no explicit trend is seen in the relative abundance of bacterial phyla along the elevation gradient (Figure 3A). Acidobacteria and Proteobacteria were the dominant bacterial phyla in all subgroups and their relative abundance showed an increasing trend with increasing altitude. Actinobacteria in general showed higher abundance toward lower altitude zones. Similarly, Ascomycota and Basidiomycota were the dominant fungal phyla in all subgroups. Basidiomycota showed an absolute dominance (52.4%) at the highest altitude zone (Figure 3B).

The relative abundance of the bacterial genera showed a marked difference in the low altitude zones as compared to the high-altitude zones. In general, the relative abundance of the bacterial genera decreased with increasing altitude. The genus *Arthrobacter* showed a remarkable presence in all the altitudinal zones, and it was most dominant in the 900-m zone along with the genus *Tumebacillus*. The genus *Massilia* dominated in the 1,200-m zone (Supplementary Figure S7A). In contrast to the bacterial genera, the relative abundance of the fungal genera did not change with the altitude. It seemed that the genus *Archaeorhizomyces* preferred altitude zones > 1,400 m, whereas the genus *Hygrocybe* preferably is more abundant in the altitude zones > 1,500 m. Furthermore, the genus *Saitozyma* favors altitudes < 1,800 m, whereas the genus *Penicillium* dominated the 1,200-m zone followed by the 900-m zone. Moreover, the genera *Russula* and *Coprinellus* dominated the 2,700-m altitude zone (Supplementary Figure S7B).

The α-diversity analysis revealed that both the Chao1 and Shannon’s index were highest for the altitudinal zone of 2,200 m in the case of bacterial communities. However, these diversity indices were found to be highest in the altitudinal zone of 900 m for fungal communities (Figure 3C). The bacterial and the fungal communities had the highest number of OTUs in the 1,400-m and 1,800-m altitudes, respectively, with a total of 2,869 OTUs for bacteria and 453 OTUs for fungi (Supplementary Figure S8). Besides, the β-diversity analysis through the NMDS test revealed that both bacterial and fungal communities could not be grouped into distinct clusters based on the altitude variations (Supplementary Figure S9). The Anosim test for 16S rRNA showed that the highest difference in the bacterial community structure is exhibited between the 900-m and 1300-m altitude zones (*R* = 0.6716, *p* = 0.003) followed by the 900-m and 2,200-m zones (*R* = 0.4056, *p* = 0.003) (Supplementary Table S2). Similar results were shown by the MRPP test. The Anosim test for ITS showed that the highest difference in the fungal community structure is exhibited between altitude zones 1,400 and 2,100 m (*R* = 0.3586, *p* = 0.001) followed by 1,400 and 2,100 m (*R* = 0.3315, *p* = 0.001). Similar results were shown by the MRPP test.

Based on the UPGMA clustering, it was observed that the bacterial community profile at the altitude zone of 900 m is much more distinct as compared to the other altitude zones, followed by the 1,200-m zone. The altitude zones from 1,500 to 2,100 m share a structural similarity in bacterial communities, whereas the altitude zones of 2,300 and 2,700 m looked similar. This indicated that the community profile at the upper extreme (2,300 and 2,700 m) and lower extreme (900 and 1,200 m) altitudinal zones are distinct from the intermediary zones, which tend to be relatively similar (Supplementary Figure S10A). Similar results were shown for the fungal community structure in which the 2,700-m zone was most distinct from other zones. The altitude zones of 900, 1,200, and 1300 m formed their sub-cluster, so they tend to be similar, but distinct from others. Likewise, the altitude zones from 1,400 to 2,300 m formed a single group (Supplementary Figure S10B).

### Community Structure and Diversity in Response to Variations in Geographical Locations

**FIGURE 4:**
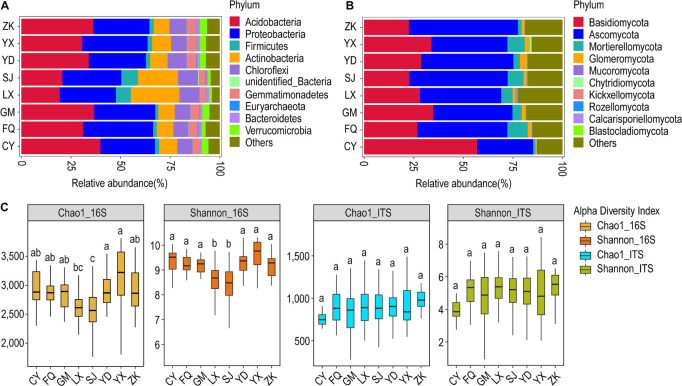
Relative abundance and α-diversity indices of soil bacterial and fungal communities in different region groups. **(A)** Relative abundance of bacteria at the phylum classification level; **(B)** relative abundance of fungi at the phylum classification level. **(C)** Chao1 and Shannon indices of bacteria and fungi. Different lowercase letters indicate significant differences between subgroups (*p* < 0.05). CY, Cang Yuan; GM, Geng Ma; ZK, Zhen Kang; LX, Lin Xiang; SJ, Shuang Jiang; FQ, Feng Qing; YD, Yong De; YX, Yun Xian.

According to the geographical distribution of the sampling sites, we analyzed ancient tea plantations located in eight counties. At the phylum classification level, the relative abundance of soil bacterial and fungal communities in tea plantations in different regions did not change significantly, but there were some differences in their relative abundance composition. Based on the geographic locations, the bacterial community was dominated by Acidobacteria and Proteobacteria at all sites. The relative abundance of Acidobacteria was highest in Cang Yuan (CY) followed by Geng Ma (GM) and Zhen Kang (ZK), whereas it was least abundant in Lin Xiang (LX) and Shuang Jiang (SJ). Interestingly, LX and SJ host a higher abundance of Actinobacteria and Chloroflexi in comparison to other locations (Figure 4A). The fungal community was dominated by Ascomycota and Basidiomycota, with the former being most abundant in CY and the latter being most abundant in ZK (Figure 4B).

At the genus level, the LX and SJ were most dominated by the bacterial genera *Arthrobacter, Bacillus*, and *Kitasatospora.* These two sites showed distinct bacterial community profiles in comparison to others. The genus *Tumebacillus* was also more abundant in these sites. However, the fungal community profile showed a distinct pattern across all the sites. CY was occupied mostly by *Amphinema* and *Inocybe. Archaeorhizomyces* formed a substantial proportion of the fungal community in other sites, with the most dominant in ZK. *Hygrocybe* was the least frequent in ZK but formed a substantial part in other sites. Besides, *Mortierella* also formed a substantial proportion in all sites except CY and ZK. *Russula* appeared to be a trademark genus in YK and SJ, whereas *Penicillium* seemed to be a hallmark genus of SJ (Supplementary Figure S11).

The bacterial α-diversity analysis revealed the highest average Chao1 value for Yun Xian (YX) and the lowest for LX and SJ; other sites have intermediary values of Chao1. Similar results were obtained for Shannon’s diversity index. The fungal α-diversity analysis revealed the highest average Chao1 value for ZK and lowest for CK; others showed intermediary average Chao1 values (Figure 4C). The highest number of OTUs for the bacterial (4,389) and fungal (1,564) communities were observed for SJ and LX, respectively (Supplementary Figure S12). The β-diversity analysis through the non-metric NMDS test did not cluster the bacterial and fungal communities according to geographical locations (Supplementary Figure S13). The Anosim test on bacterial communities revealed that LX was quite distinct from all other sites. It differed most from Feng Qing (FQ) (*R* = 0.5371, *p* = 0.001) followed by CY (*R* = 0.5015, *p* = 0.001), whereas it showed least difference with YX (*R* = 0.3685, *p* = 0.001). The MRPP test also revealed LX distinct from all other sites. In the case of fungal communities, the Anosim test revealed CY to be most distinct from all others, followed by LX (Supplementary Table S3).

In the case of bacteria, based on the UPGMA clustering results, LX and SJ formed one cluster, whereas a separate cluster was formed by other locations, with ZK and Yong De (YD) forming their sub-cluster. This indicated that LX and SJ have similar community structures and are quite distinct from other sites. In the case of fungi, two main sub-clusters were formed. GM, LX, and SJ formed one group, whereas YD and YX formed another group. Other sites are segregated into their leaves. This indicated that CY is the most distinct plantation site from others in terms of the fungal community, followed by ZK. The LX, and SJ as well as YD and YX are much similar to each other (Supplementary Figure S14).

### Correlation Analysis

**FIGURE 5:**
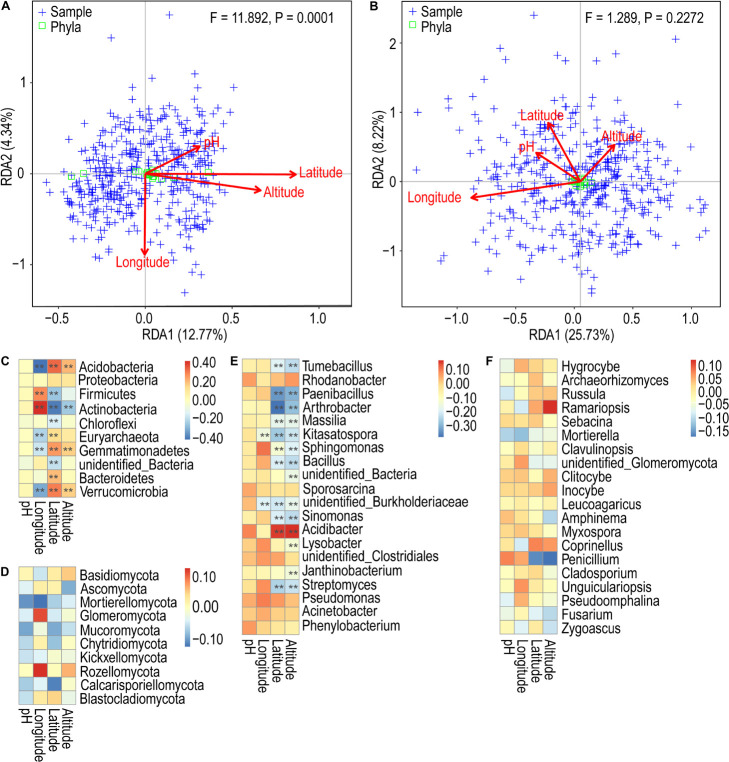
Redundancy analysis of the composition of soil bacterial **(A)** and fungal **(B)** communities in tea plantations with environmental and geographical factors. Spearman’s correlation analysis between phylum/genus of relative abundance and environmental and geographical factors. **(C)** Bacterial phylum classification level; **(D)** fungal phylum classification level; **(E)** bacterial genus classification level; **(F)** fungal genus classification level. **p < 0.01.

Redundancy analysis showed that the first two axes explained only 17.11 and 33.95% of the variance. From Figure 5A, it is evident that the environmental factors altitude and latitude exhibit highly positively correlated bacterial community profiles, followed by latitude and pH, and altitude and pH. In contrast, the environmental factors pH and longitude exhibit negatively correlated community profiles. The variables latitude and longitude do not correlate. In the case of fungi, the environmental factors pH and latitude have a highly positively correlated community profile, followed by latitude and altitude. In contrast, the environmental factors altitude and longitude exhibit negatively correlated community profiles. The variables pH and latitude and do not correlate (Figure 5B). Mantel test revealed very weak correlations between the variables and the bacterial and fungal community composition (Table 1). However, altitude and latitude appeared to be relatively more effective. Furthermore, Spearman correlation analysis was used to determine the relationship between the relative abundance of bacterial and fungal communities and environmental and geographic factors. We observed that Proteobacteria did not have any significant correlation with any variable. Furthermore, pH showed a very weak or no correlation with all the variables. Longitude, altitude, and latitude showed a significant positive or negative correlation with all or some phyla (Figure 5C). At the genus level, the strongest significant correlations were shown by altitude and latitude (Figure 5E). In the case of fungal phyla and genera, significant correlations were lacking (Figures 5D,F).

**TABLE 1:** Mantel-test analysis of environmental and geographical factors with bacterial and fungal community composition.

Environmental and geographical factors	Bacterial		Fungal	
	*R*	*P*	*R*	*P*
pH	–0.0084	0.6757	–0.0084	0.6840
Lo	–0.0013	0.5254	–0.0013	0.5236
La	0.0354	0.0169*	0.0354	0.0166*
Al	0.0406	0.0047**	0.0406	0.0044**
Lo + La	0.0196	0.1345	0.0196	0.1389
Lo + Al	0.0061	0.3712	0.0061	0.3690
La + Al	0.0441	0.0044**	0.0441	0.0070**
Lo + La + Al	0.0220	0.1074	0.0220	0.1049
pH + Lo + La + Al	0.0113	0.2685	0.0113	0.2825

Lo, Longitude; La, Latitude; Al, Altitude. *P < 0.05; **P < 0.01.

## Discussion

In the soil–plant rhizobiome, the most active and decisive soil microorganisms play an active role in the exchange of matter and energy, soil formation and development, plant–soil interactions, etc. (Urbina et al., 2018; Tan et al., 2019). The suitable soil pH for the growth of tea plants is 4.00–6.50, with the most optimum being 4.50–5.50. Recent studies have revealed that in China, the soil is constantly becoming acidic for the tea plantation sites, with pH as low as < 4.50 at many sites (Guo et al., 2010; Yan et al., 2020). Our analysis also revealed an acidic pH (4.68) of ancient tea plantations in Lincang city, which is further corroborated by Yan et al. (2020). Although not more acidic than the lower optimal value (4.00), the situation has reached a critical level for tea plantations in Lincang city, which would lead to a serious reduction in the growth and survival rate of tea plants, thereby affecting the yield.

The microbial diversity of the tea plantation sites not only varies with wastelands and woodlands but also with the age of different tea plantation sites (Xue et al., 2007, 2008). The intensity and duration of tea planting have a significant impact on the microbial community structure, biomass, and its function (Han et al., 2007). Relatively high community diversity was observed in 50-year-old tea plantations (Xue et al., 2006). Thus, tea plantation monoculture, tea plant age, different management patterns, and land use types have a significant impact on soil microbial community diversity, composition, and structure. Our study showed that Acidobacteria and Proteobacteria (Alpha, Delta, and Gamma) were the dominant bacterial phyla in all the tea plantation sites, whereas Ascomycota and Basidiomycota were the dominant fungal phyla (Supplementary Figure S15). With some minor differences, our findings were corroborated by several studies (Zhao et al., 2012; Li Y. C. et al., 2016; Arafat et al., 2019; Tan et al., 2019). Acidobacteria are particularly abundant in soils with very low resource utilization (Quaiser et al., 2003; Fierer et al., 2007). Alphaproteobacteria can grow at very low nutrient levels and can induce nitrogen fixation with plants. Gammaproteobacteria are associated with large amounts of available nutrients and control disease inhibitory activity through non-ribosomal peptide synthase (Mendes et al., 2011; Li Y. C. et al., 2016). Deltaproteobacteria are an important contributor to the anaerobic process of the sulfur cycle. Ascomycota promotes the decay of animal and plant remains in the soil (Mendes et al., 2011), whereas basidiomycota forms mycorrhiza with roots of plants. Previous studies have confirmed the presence of *Bacillus* within the rhizobiome of tea plants, with *B. subtilis* and *B. mycoides* forming a major part of the bacterial population, even in unfavorable times. *Bacillus* sp. exhibit antagonistic activity against fungal isolates; they inhibit the growth of fungal mycelium and cause structural abnormalities in the rhizosphere of tea plants (Pandey and Palni, 1997; Singh et al., 2007). The abundance of *Bradyrhizobium*, *Mycobacterium*, and *Sphingomonas* gradually decreases with the increasing age of tea plantation sites, whereas the *Granulicella* shows a reverse trend. Details about their functions are lacking; however, *Bradyrhizobium* is essential in promoting plant growth (Antoun et al., 1998; Li Y. C. et al., 2016). Our study confirmed the presence of the genus *Bacillu*s only which occupied a certain percentage of all sampled sites.

Due to the monoculture of tea plants, enhanced use of chemical fertilizers (especially nitrogen fertilizers), and accumulation of organic matter, the soil acidification of tea plantation sites is deepening. This may lead to a significant reduction in microbial species within the soil (Wilson et al., 2004; Abe et al., 2006; Ruan et al., 2007; Li S. Y. et al., 2016; Yan et al., 2018). Although the forest and tea plantation soils have similar acidic pH, their microbial community structures are different, suggesting that the differences are not solely due to variations in pH (Yao et al., 2000). Koga et al. (2003) showed that in acidic tea plantation soils, the activity of soil microbes showed a tendency to rise first and then decrease with increasing pH. The application of lime can control the acidification of tea plantation soils. It has a significant effect on the structure and diversity of the microbial community in the soil, as the diversity increases with increasing lime concentration (Xue et al., 2010). However, a study by Wilson et al. (2004) found that soil microorganisms are usually not affected by soil pH. Our study found that the composition and structure of soil bacterial and fungal communities exhibited a mono-peak pattern with pH variation. The relative abundance of the fungi (pH = 4.00–5.50) was more significantly affected by pH than that of the bacteria (pH = 4.50–5.50), whereas the fungal diversity was less correlated with pH than that of the bacteria. Similar results were obtained during soil pH studies on tea plant fitness (Ruan et al., 2007; Guo et al., 2010; Yan et al., 2020). Although the bacterial phyla did not show any significant trend along the pH gradients; however, Actinobacteria were found to be less abundant at low pH, whereas Acidobacteria exhibited higher abundance at higher pH. In contrast, the fungal phyla Ascomycota and Basidiomycota showed a clear declining trend with increasing pH, whereas Mortierellomycota exhibited higher abundance with increasing pH. The α-diversity analysis revealed that the bacterial community composition is more diverse at a pH range of 4.50–5.00, whereas the fungal diversity remained relatively same across different pH gradients. Our study showed that the pH range of 4.50–5.00 seemed more optimal, as the number of OTUs was found to be highest in this range for both bacteria and fungi. Besides, the α-diversity of the rhizosphere bacterial community was positively correlated with pH in perennial shrubs *Caragana* spp., with pH as a significant factor affecting both the α-diversity and richness (Na et al., 2018). Li Y. C. et al. (2016) found that only *Granulicella* in Acidobacteria increased significantly with decreasing pH in tea plantation soil, whereas the relative abundance of Acidobacteria was not significantly correlated with soil pH. Further studies showed that the soil bacterial communities are usually closely related to soil pH and their relative abundance and diversity are positively correlated with pH, a pattern that applies to both the overall bacterial community composition and the composition of individual bacterial communities. However, for fungi, the relative abundance is not affected by pH and the diversity is only weakly correlated with pH, suggesting that tea plantation ecosystems have some similarities and specificities compared to others, such as agroecosystems (Jones et al., 2009; Rousk et al., 2010; Li Y. C. et al., 2016). Besides, our study found that *Archaeorhizomyces* was the dominant fungal genus in all subgroups and had a higher relative abundance in the low pH gradient group. Sequence characteristics indicate that *Archaeorhizomyces* occupy plant root intervals in deep soils with low pH and high nutrient transformation, and their relative abundance is significantly correlated with the fungal plant pathogen *Typhula*, the decaying fungus *Exophiala*, the *Suillus* exophytic root family, and *Tubeufia* and *Omphalotus*, which are all involved in the decay process of vegetation (Carrino-Kyker et al., 2016; Choma et al., 2016; Pinto-Figueroa et al., 2019). When soils become acidic, the soil microorganisms have to elicit a physiological response to cope with decreasing pH like synthesis of protective membrane proteins and fatty acids, production of buffer molecules to maintain internal pH homeostasis, and pumping of H^+^ ions to maintain electoral gradient across the membrane. Besides, low pH is associated with low soil inorganic phosphorous (Pi) due to mobilized aluminum (Al). Soil microorganisms overcome this low Pi by acquiring more organic P (Po). Increase in soil pH altered both bacterial and fungal communities in temperate deciduous forests (Carrino-Kyker et al., 2016).

Studies on the relationship between microbial community profile and altitude vary. Some studies establish an inverse relationship between soil microbial diversity and altitude (Tian et al., 2017; Li et al., 2018; Nottingham et al., 2018). Other studies confirm that there is no clear-cut relationship between them and different microbial groups show different patterns along the altitude gradients (Fierer et al., 2011). Our analysis showed an increase in abundance of Acidobacteria and Proteobacteria across the different altitudinal zones, whereas Actinobacteria were abundant at lower altitudes. In case of fungi, Basidiomycota was the most abundant at higher altitudes. In general, the abundance of bacterial genera increased with increasing altitude, whereas the fungal genera did not reveal any recognizable variation. The diversity of the bacterial and the fungal communities was highest at an altitude of 2,200 and 900 m, respectively. Acidobacteria were found to decrease monotonically with increasing altitude (Bryant et al., 2008), whereas a monotonic increase was observed for the bacteria by Wang et al. (2011). Both the single peak pattern (bacteria) and the bimodal pattern (archaebacteria) were observed for the microbial diversity (Singh et al., 2012). The nitrogen-fixing bacteria also showed diverse relationships with altitude (Wang et al., 2019). According to Shen et al. (2015), the composition of the bacterial communities at high altitudes is most complex and diverse among all microbes. In the case of fungi, the abundance generally decreases with increasing altitude, but there is no significant correlation between its uniformity and altitude. Our study revealed a single peak pattern of bacterial abundance with increasing altitude and a double peak pattern of bacterial diversity with increasing altitude. However, a double peak pattern was observed for both the fungal abundance and fungal diversity with increasing altitude. These results indicate that the diversity, community composition, and structure of bacteria and fungi in tea plantation soil are somewhat different and show different patterns of altitude distribution. Furthermore, *Arthrobacte*r showed a higher relative abundance at lower altitudes in comparison to higher altitudes, whereas *Penicillium* showed an absolute predominance at lower altitudes. This is corroborated by several studies (Widden, 2018; Kumar et al., 2019).

With the aim of evaluating the variation in arbuscular mycorrhizal fungi (AMF) along an altitudinal gradient in rupestrian grasslands in Brazil, Coutinho et al. (2015) found less similarity of AMF species across different altitudinal zones. Altitudinal zone with similar soil characteristics showed overlap in AMF species. The AMF species composition was different at extreme elevations and similar in intermediate zones, suggesting that the distribution of the species along different attitudes may be governed by soil variability (Coutinho et al., 2015). Similarly, altitude had a significant effect on the composition of the soil fungal communities associated with Norway spruce (*Picea abies*), whereas the age of the tree governs community diversity (Schön et al., 2018). Besides, altitude significantly impacts the relative abundances of the dominant phyla and classes of soil fungi and the interface of altitude and season affects the relative abundances of Ascomycota and Basidiomycota (Ji et al., 2021). While evaluating the variations in soil microbial community diversity and structure along five climate zones (from subtropical to cold temperate), with an altitude of 1,600–3,200 m across the eastern slope of Mount Gongga, Yang and Wu (2020) found that the soil bacterial but not fungal diversity changed across the vertical climate zones, with highest bacterial diversity occurring in subtropical and cold temperate zones. The fungal (composed of Ascomycota, Basidiomycota, and Zygomycota) and bacterial (composed of Proteobacteria, Acidobacteria, Actinobacteria, and Bacteroidetes) relative abundance enhanced with increasing altitude. No significant shifts in abundance or community composition were detected for archaeal community with altitude (Siles and Margesin, 2016). Thus, our results of altitudinal impact on tea-associated soil bacterial and fungal communities may be indirectly governed by physiochemical properties of the soil, seasonal effect, age of the tea plants, and interaction between altitude and climate along an altitudinal gradients.

In general, the relative abundance of both bacterial and fungal phyla did not change significantly with geographical locations. Acidobacteria and Proteobacteria were dominant bacterial phyla and Ascomycota and Basidiomycota were dominant fungal phyla at all the eight counties. The locations SJ and LX were peculiar for bacterial communities as they contained highest abundance of Actinobacteria and Chloroflexi, and *Arthrobacter, Bacillus*, and *Kitasatospora*. However, these sites have the lowest bacterial diversity. For fungi, CY and ZK were most peculiar with the former showing dominance of Ascomycota, *Amphinema*, and *Inocybe*, and the latter showing dominance of Basidiomycota and *Archaeorhizomyces.* ZK also showed the highest fungal diversity.

The environmental and geographic factors that sustain the soil microbes in tea plantations are important drivers of the composition and structure of soil microbial communities. Previous studies have found that the soil pH at tea plantation sites is a key factor influencing the bacterial and fungal community structure besides NO3-N, SOC, TOC SOM, and AP (Li Y. C. et al., 2016; Arafat et al., 2019; Tan et al., 2019). Zhao et al. (2012) found that based on limited data, relationships between environmental factors (pH) and tea plantation soil bacterial communities cannot be derived, and observed that the more alike the environmental variables are, the more similar is the bacterial community structure. Based on our results, we obtained somewhat similar patterns of bacterial and fungal communities across the environmental and geographic factors, with only a few phyla and genera showing a significant variation with these factors. Furthermore, Mantel test and Spearman correlation analyses revealed that latitude and altitude were more pronounced for the composition and structure of the bacterial communities (both at the phylum classification level and genus classification level) than those of the fungi, although the correlation was generally weak. Moreover, the analysis of the relative abundance and diversity (Supplementary Figure S16) of the bacterial and fungal communities in tea plantations distributed in anthropogenic versus inactive areas did not show any difference (Supplementary Figure S16).

The tea plantation sites have been under the management and selection of tea farmers for thousands of years. The composition and structure of the soil microbial community, and the correlation between its abundance and environmental and geographical factors have been artificially domesticated. The interference of anthropogenic activities is the main factor governing the fungal community selection, whereas for the bacterial community, it is more selective to the environmental adaptation, rather than the adaptation to human activities (Supplementary Figure S16).

## Data Availability Statement

The datasets presented in this study can be found in online repositories. The names of the repository/repositories and accession number(s) can be found in the article/Supplementary Material.

## Author Contributions

LK, GX, YW, ZW, GL, DL, JY, SY, CW, LY, ShiZ, ShuZ, JZ, and TH collected the soil samples. GX, ZW, and CW performed the DNA extraction and quality control. GX and ZW performed the soil pH measurement. LK, GX, and ZW performed the data analysis and manuscript writing. AM, DL, WC, and JS revised and improved the manuscript. YD, LZ, HG, and JX directed the work. All authors provided ideas, participated in the evaluation of the results and discussion, read and approved the final manuscript.

## Conflict of Interest

The authors declare that the research was conducted in the absence of any commercial or financial relationships that could be construed as a potential conflict of interest.

## Publisher’s Note

All claims expressed in this article are solely those of the authors and do not necessarily represent those of their affiliated organizations, or those of the publisher, the editors and the reviewers. Any product that may be evaluated in this article, or claim that may be made by its manufacturer, is not guaranteed or endorsed by the publisher.

## Footnotes

1
http://www.fao.org/home/en/

2
http://www.stats.gov.cn/english/

3
http://www.efloras.org/

## Supplementary Material

Supplementary Figure S1 Range of mean pH distribution of tea plantations soil.

Supplementary Figure S2 Rarefaction curves of bacterial **(A)** and fungal **(B)** communities.

Supplementary Figure S3 Relative abundance of soil bacterial and fungal communities in different pH groups. **(A)** Relative abundance of bacteria at the genus classification level (first 20 bacterial genus, same below); **(B)** relative abundance of fungi at the genus classification level (first 20 fungi genus, same below).

Supplementary Figure S4 OTUs petal graph analyses of the soil bacterial **(A)** and fungal **(B)** communities in in different pH groups.

Supplementary Figure S5 NMDS analysis of soil bacterial **(A)** and fungal **(B)** communities in different pH groups.

Supplementary Figure S6 UPGMA cluster tree analysis of soil bacterial **(A)** and fungal **(B)** communities in different pH groups.

Supplementary Figure S7 Relative abundance of soil bacterial and fungal communities in different altitudes groups. **(A)** Relative abundance of bacteria at the genus classification level; **(B)** relative abundance of fungi at the genus classification level.

Supplementary Figure S8 OTUs petal graph analyses of the soil bacterial **(A)** and fungal **(B)** communities in different altitudes groups.

Supplementary Figure S9 NMDS analysis of soil bacterial **(A)** and fungal **(B)** communities in different altitudes groups.

Supplementary Figure S10 UPGMA cluster tree analysis of soil bacterial **(A)** and fungal **(B)** communities in different altitudes groups.

Supplementary Figure S11 Relative abundance of soil bacterial and fungal communities in different regions groups. **(A)** Relative abundance of bacteria at the genus classification level; **(B)** relative abundance of fungi at the genus classification level.

Supplementary Figure S12 OTUs petal graph analyses of the soil bacterial **(A)** and fungal **(B)** communities in in different regions groups.

Supplementary Figure S13 NMDS analysis of soil bacterial **(A)** and fungal **(B)** communities in different regions groups.

Supplementary Figure S14 UPGMA cluster tree analysis of soil bacterial **(A)** and fungal **(B)** communities in different regions groups.

Supplementary Figure S15 Relative abundance of soil bacterial and fungal communities in 101 ancient tea plantations. **(A)** Relative abundance of bacteria at the phylum classification level (first 10 bacterial phylum, same below); **(B)** relative abundance of fungi at the phylum classification level (first 10 fungi phylum, same below). (ATP, ancient tea plantation).

Supplementary Figure S16 Relative abundance and α-diversity indices of soil bacterial and fungal communities in human activity region and human inactive region. **(A)** Relative abundance of bacteria at the phylum classification level; **(B)** relative abundance of fungi at the phylum classification level; **(C)** Chao1 and Shannon indices of bacteria and fungi. Different lowercase letters indicate significant differences between subgroups (*P* < 0.05). (HAR, human activity region; HIR, human inactive region).

Supplementary Table S1 Significance test by Anosim and MRPP of soil bacterial and fungal community in different pH gradient groups.

Supplementary Table S2 Significance test by Anosim and MRPP of soil bacterial and fungal community in different altitudes groups.

Supplementary Table S3 Significance test by Anosim and MRPP of soil bacterial and fungal community in different regions groups.
