# Supplementary material for: Large-Scale Characterization of the Soil Microbiome in Ancient Tea Plantations Using High-Throughput 16S rRNA and Internal Transcribed Spacer Amplicon Sequencing
Source: Front Microbiol. 2021 Oct 15;12:745225. doi: 10.3389/fmicb.2021.745225 (PMC8555698; doi:10.3389/fmicb.2021.745225)
Supplement: Supplementary Figure S1 — Range of mean pH distribution of tea plantations soil. [file Data_Sheet_1.doc]

**Large-scale characterization of the soil microbiome in ancient tea plantations using high-throughput 16S rRNA and ITS amplicon sequencing**


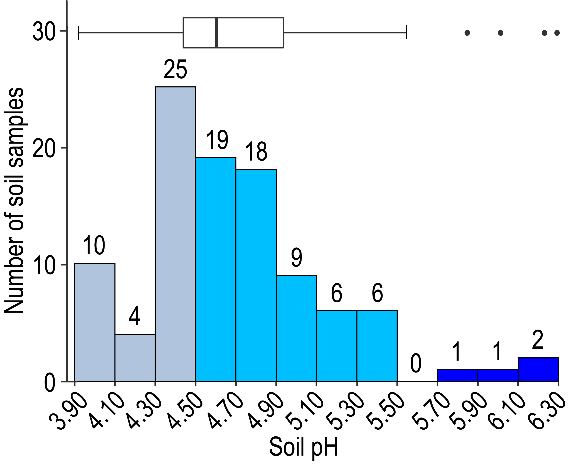


**Figure S1.** Range of mean pH distribution of tea plantations soil.


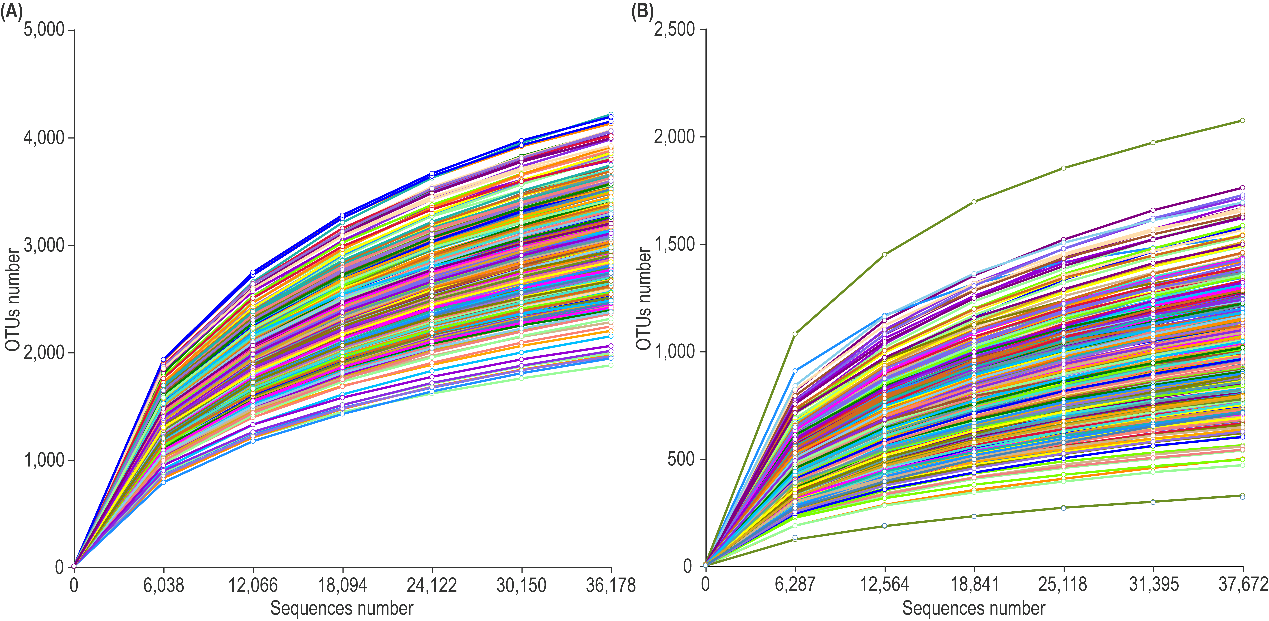


**Figure S2.** Rarefaction curves of bacterial (A) and fungal (B) communities.


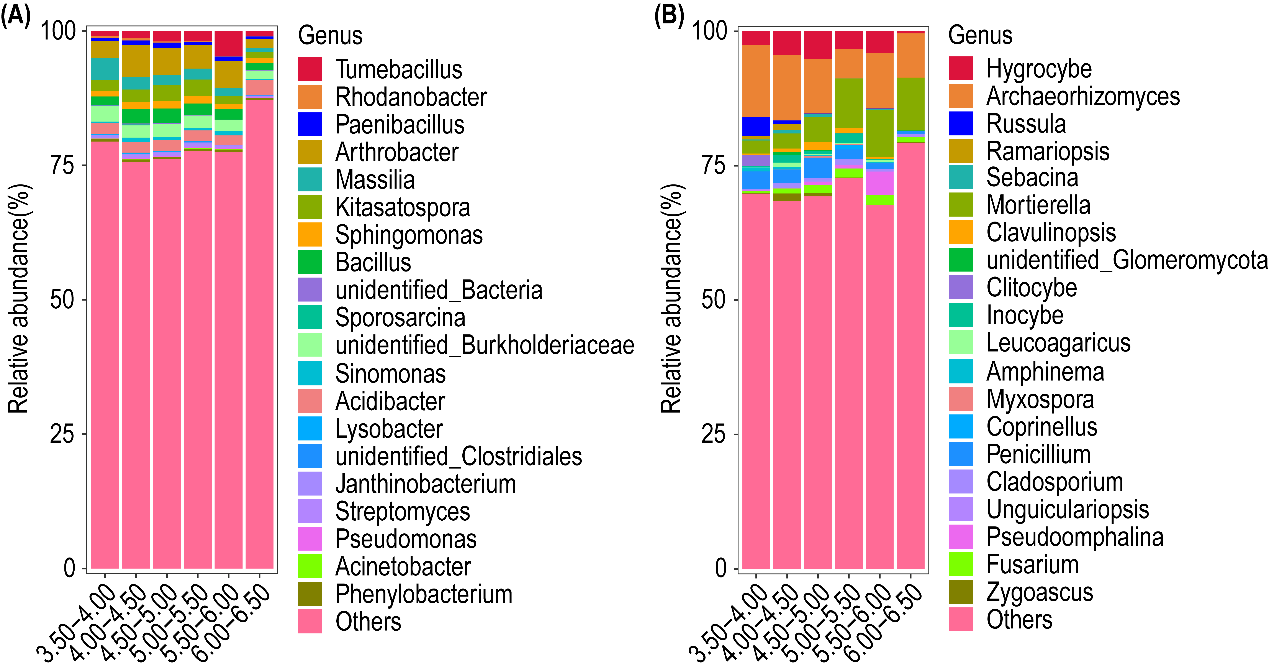


**Figure S3.** Relative abundance of soil bacterial and fungal communities in different pH groups. (A) Relative abundance of bacteria at the genus classification level (first 20 bacterial genus, same below); (B) relative abundance of fungi at the genus classification level (first 20 fungi genus, same below).


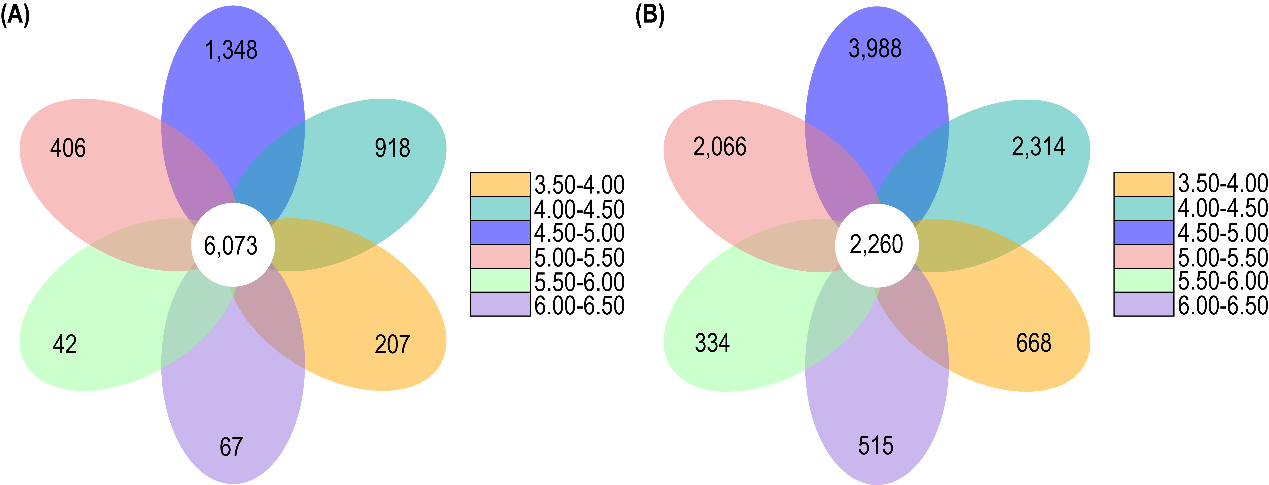


**Figure S4.** OTUs petal graph analyses of the soil bacterial (A) and fungal (B) communities in in different pH groups.


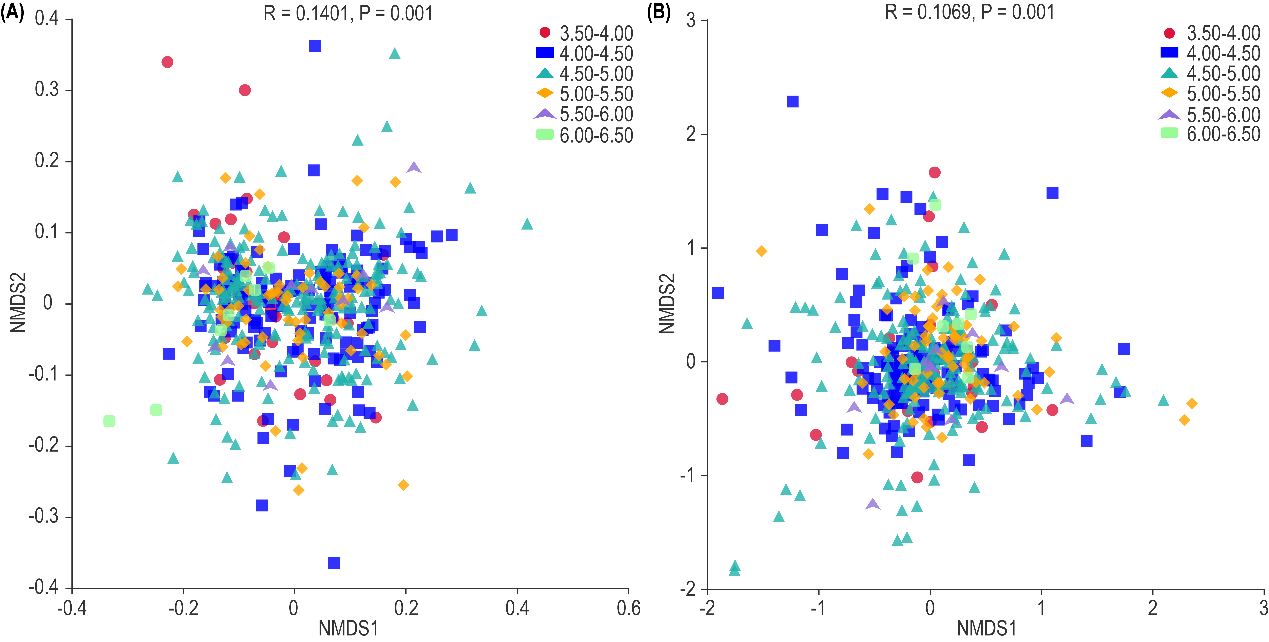


**Figure S5.** NMDS analysis of soil bacterial (A) and fungal (B) communities in different pH groups.


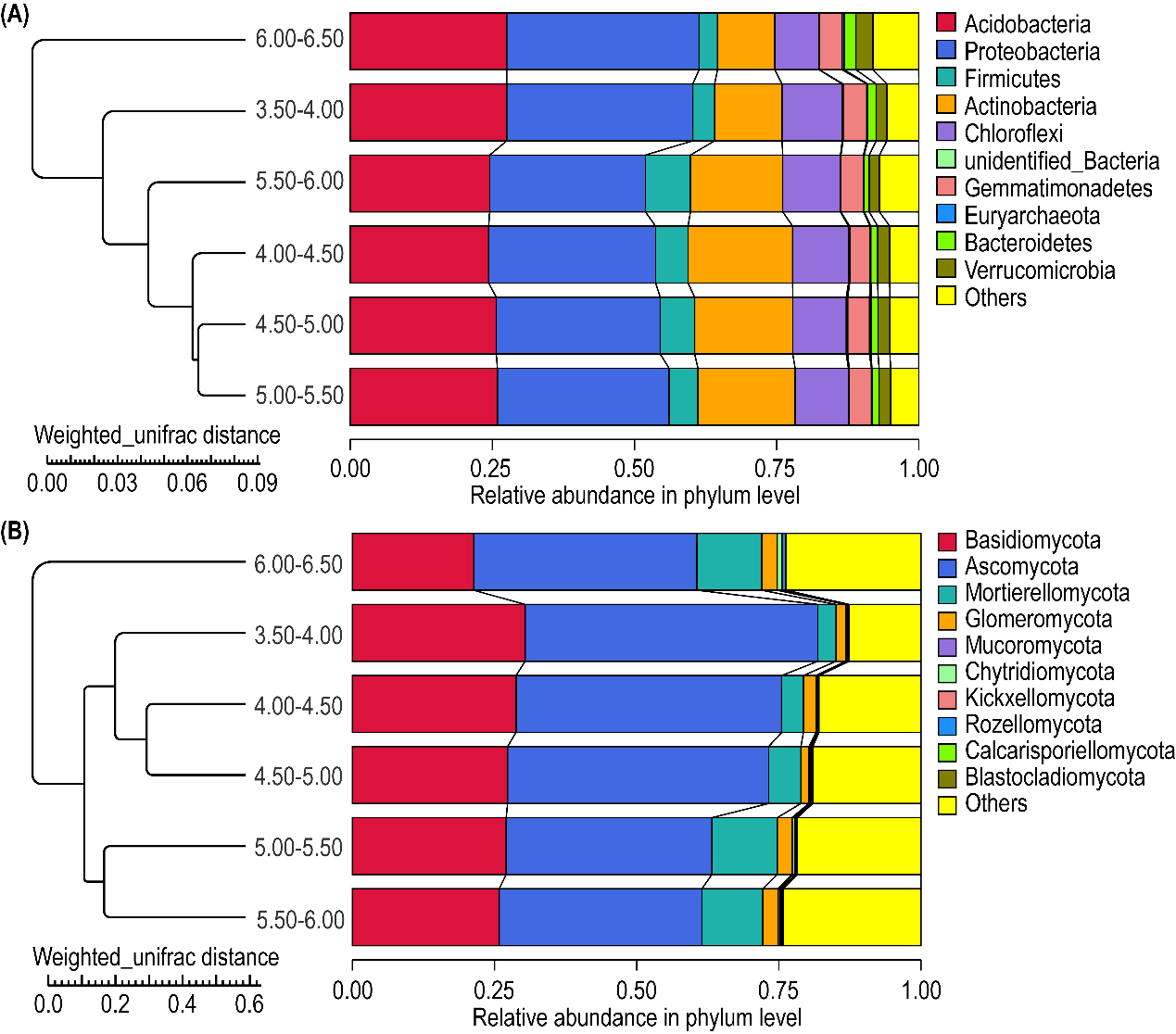


**Figure S6.** UPGMA cluster tree analysis of soil bacterial (A) and fungal (B) communities in different pH groups.


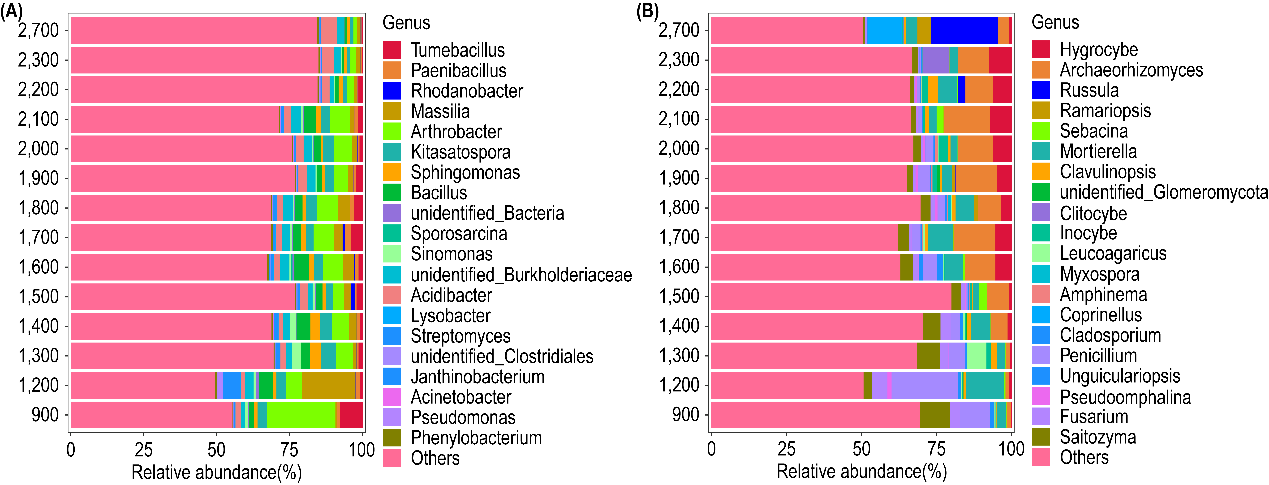


**Figure S7.** Relative abundance of soil bacterial and fungal communities in different altitudes groups. (A) Relative abundance of bacteria at the genus classification level; (B) relative abundance of fungi at the genus classification level.


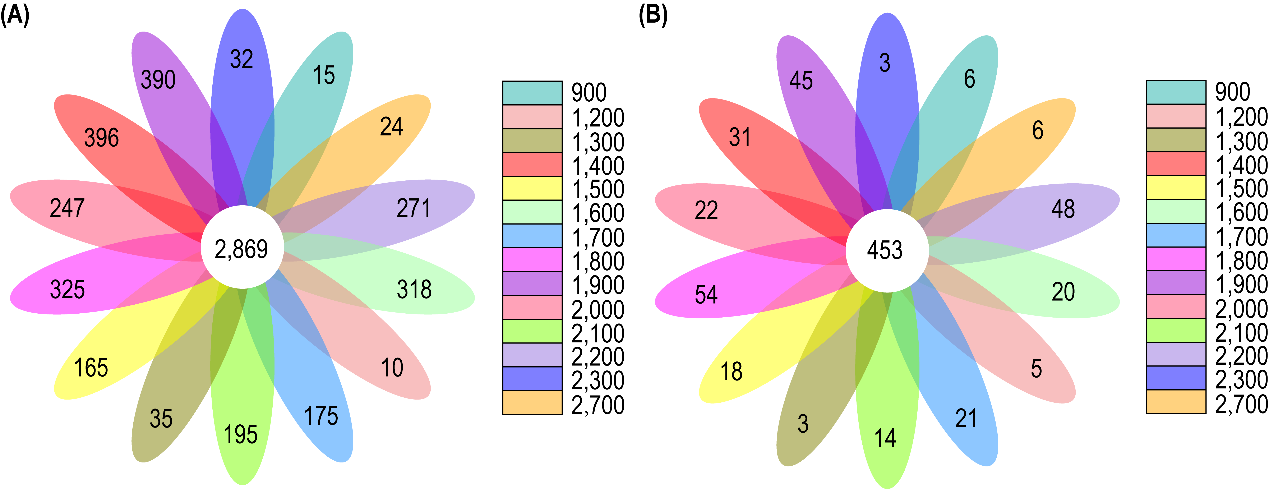


**Figure S8.** OTUs petal graph analyses of the soil bacterial (A) and fungal (B) communitiesin in different altitudes groups.


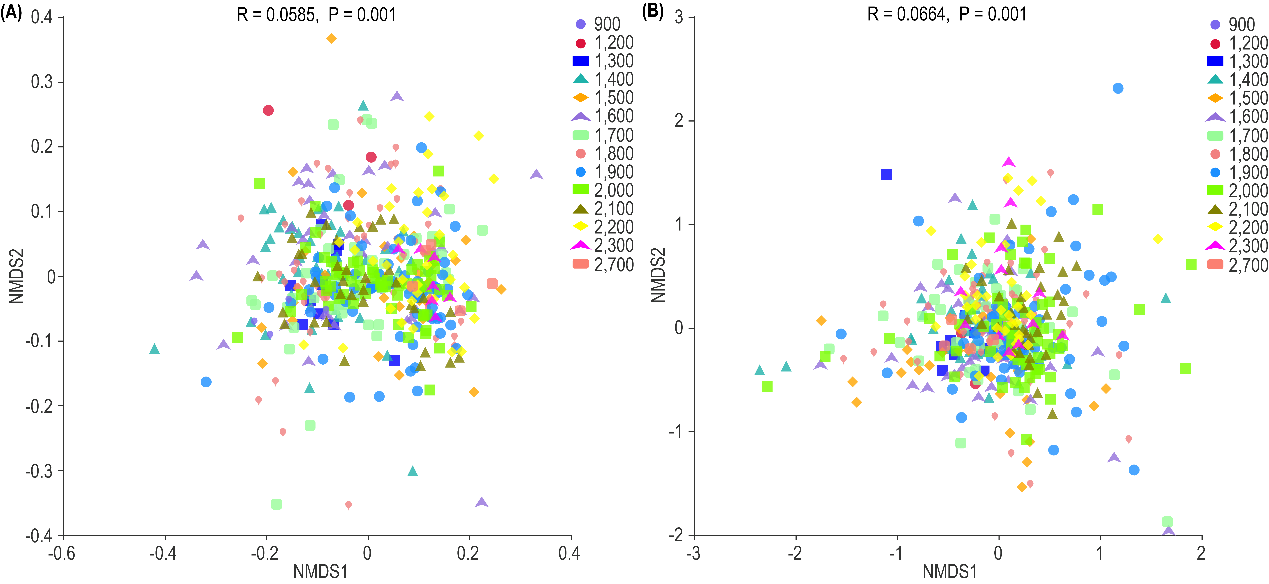


**Figure S9.** NMDS analysis of soil bacterial (A) and fungal (B) communities in different altitudes groups.


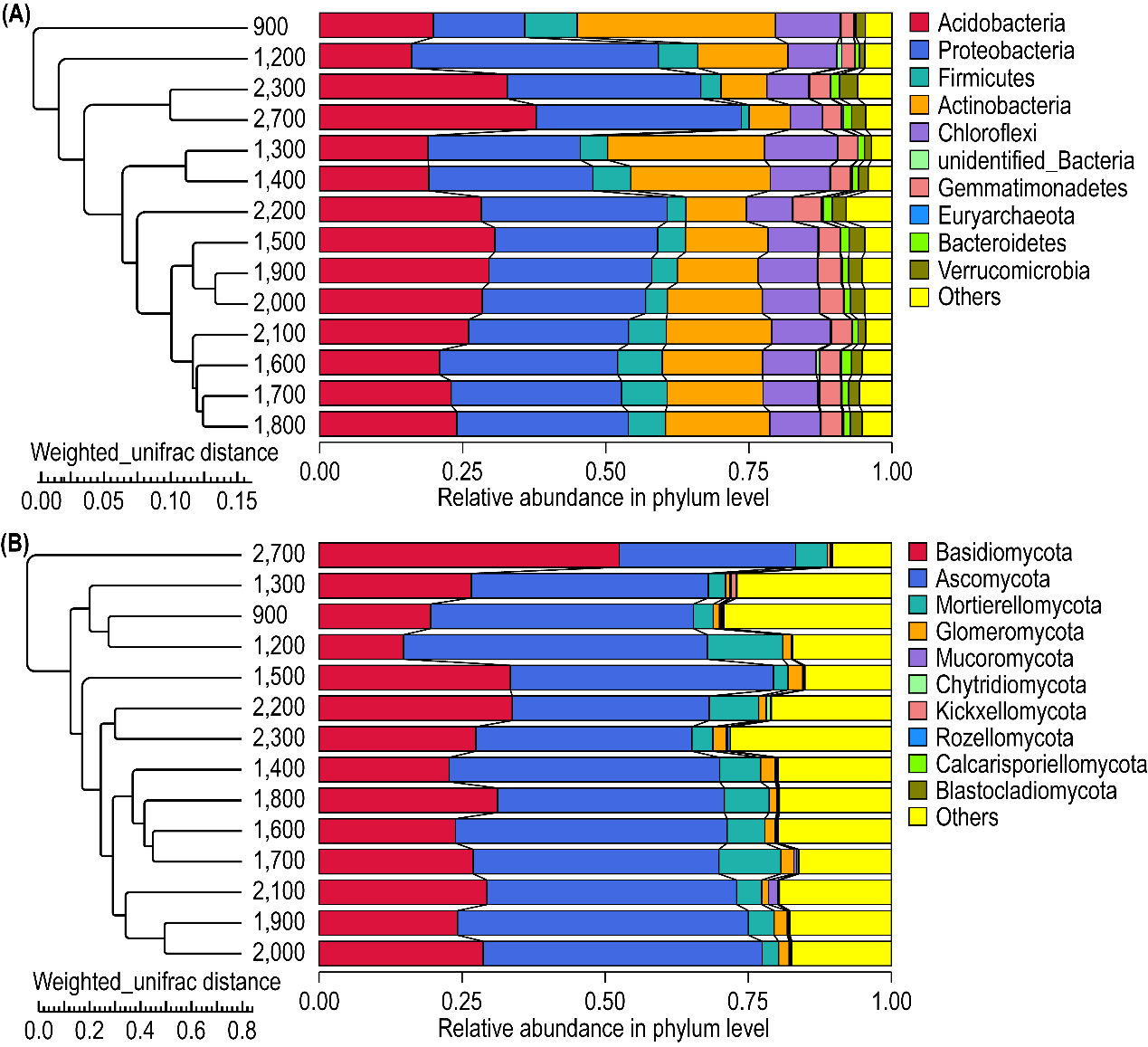


**Figure S10.** UPGMA cluster tree analysis of soil bacterial (A) and fungal (B) communities in different altitudes groups.


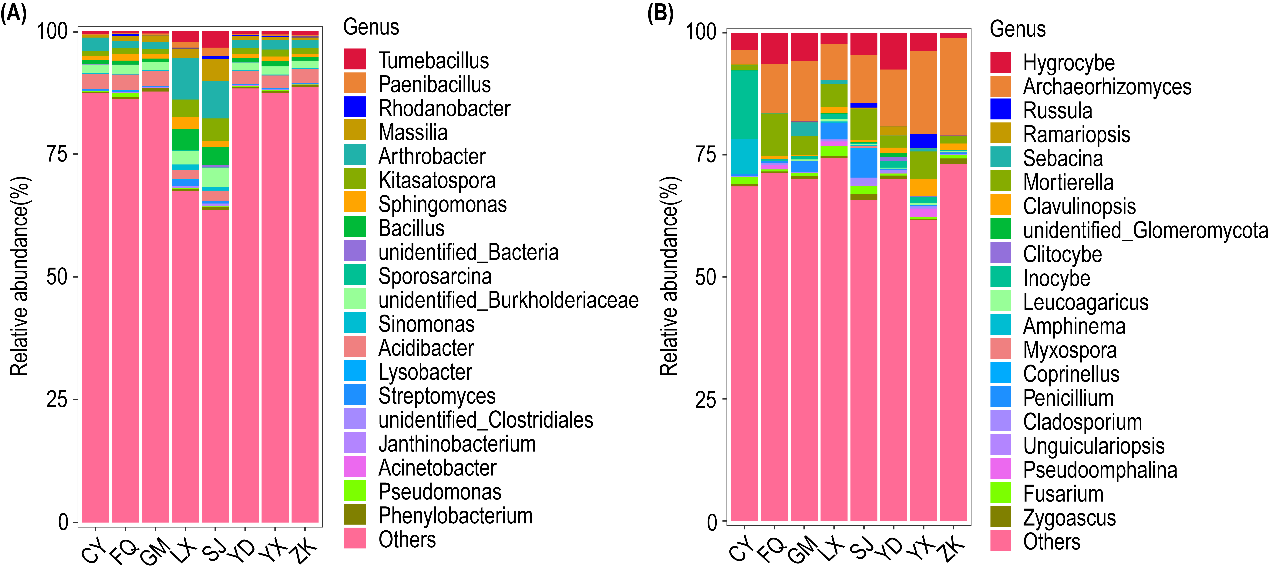


**Figure S11.** Relative abundance of soil bacterial and fungal communities in different regions groups. (A) Relative abundance of bacteria at the genus classification level; (B) relative abundance of fungi at the genus classification level.


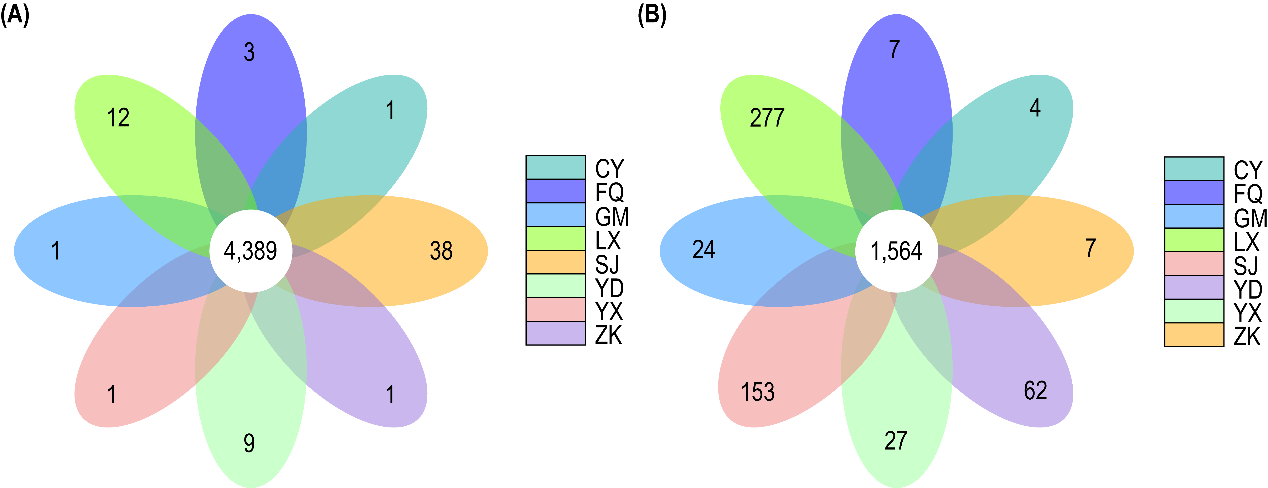


**Figure S12.** OTUs petal graph analyses of the soil bacterial (A) and fungal (B) communities in in different regions groups.


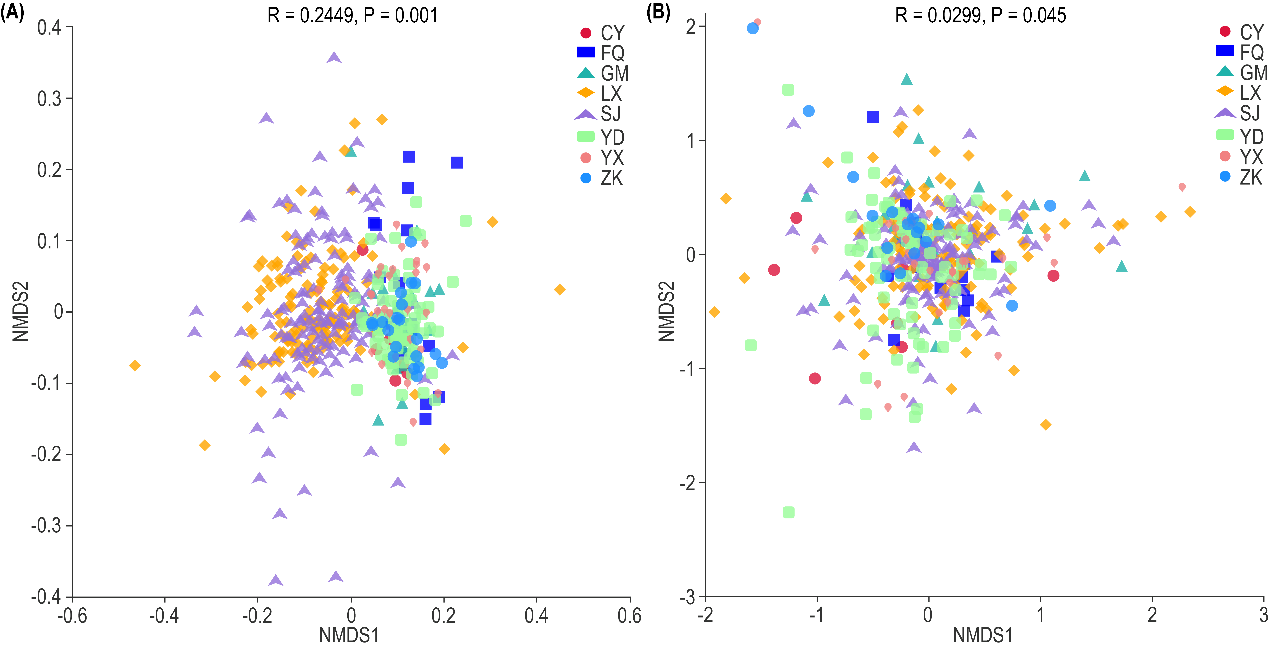


**Figure S13.** NMDS analysis of soil bacterial (A) and fungal (B) communities in different regions groups.


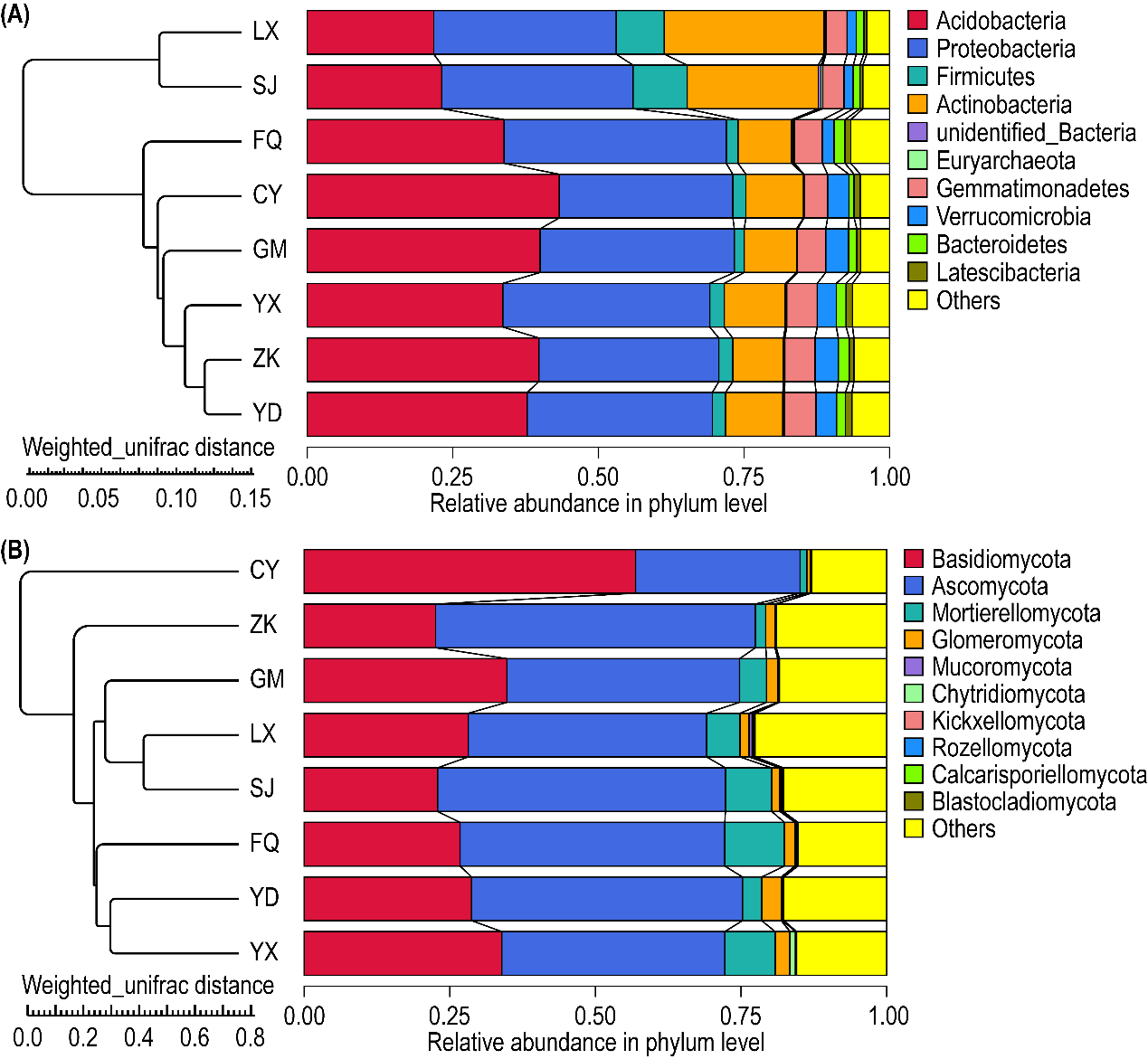


**Figure S14.** UPGMA cluster tree analysis of soil bacterial (A) and fungal (B) communities in different regions groups.


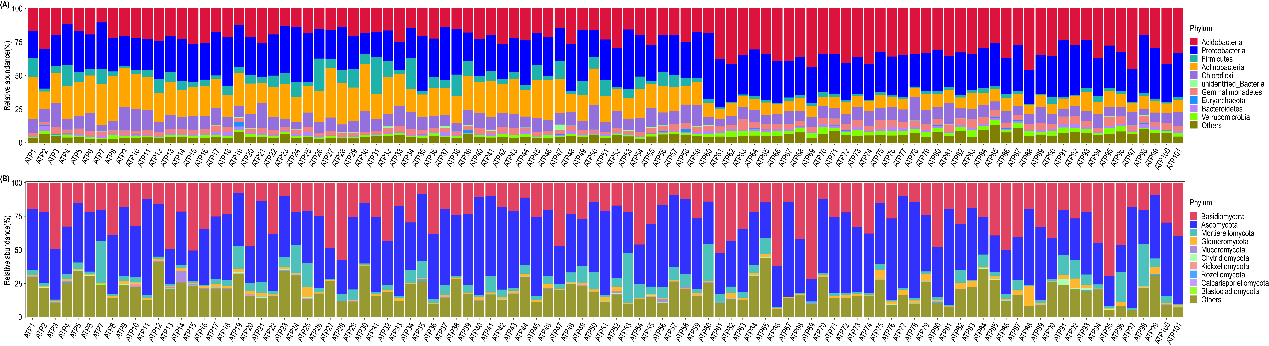


**Figure S15.** Relative abundance of soil bacterial and fungal communities in 101 ancient tea plantations. (A) Relative abundance of bacteria at the phylum classification level (first 10 bacterial phylum, same below); (B) relative abundance of fungi at the phylum classification level (first 10 fungi phylum, same below). (ATP: ancient tea plantation).


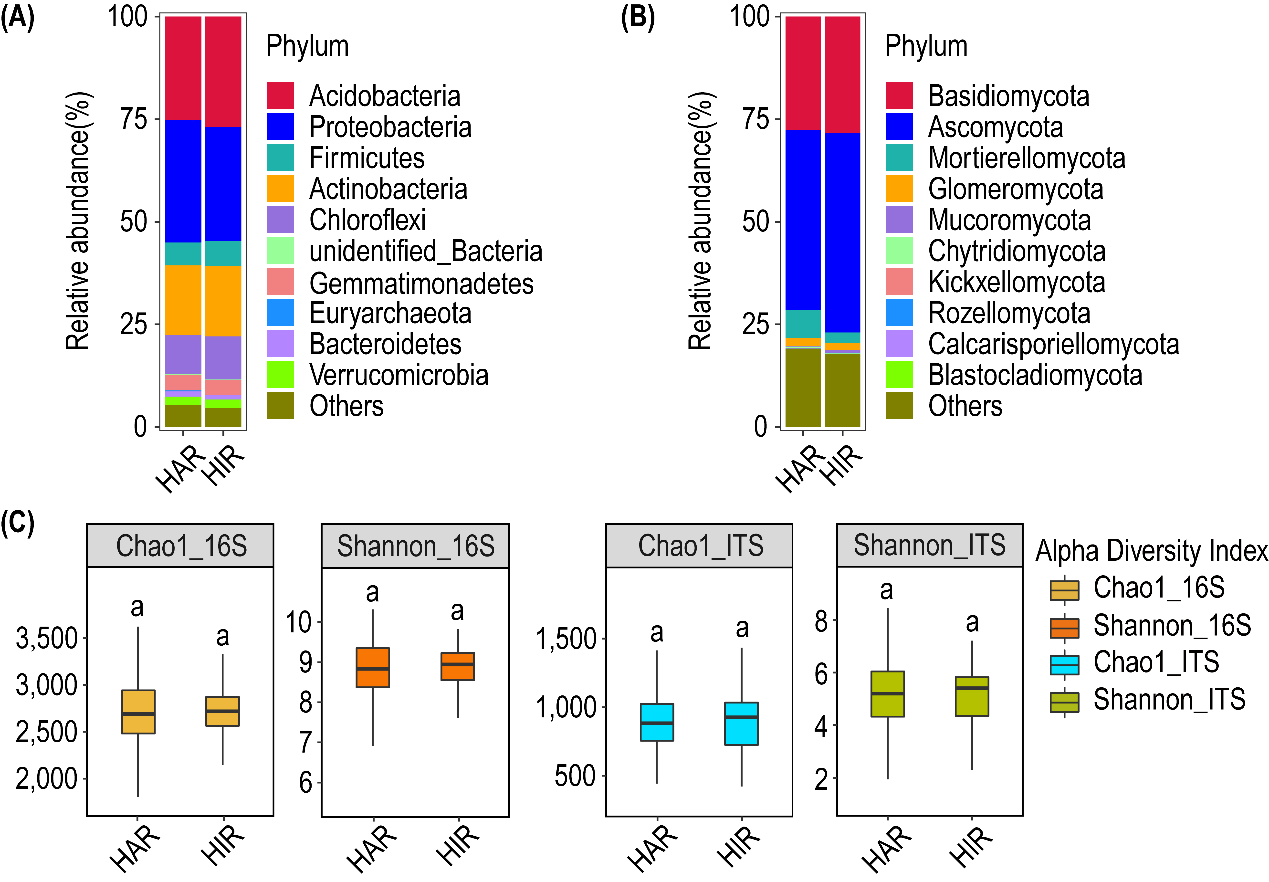


**Figure S16.** Relative abundance and α-diversity indices of soil bacterial and fungal communities in human activity region and human inactive region. (A) Relative abundance of bacteria at the phylum classification level; (B) relative abundance of fungi at the phylum classification level; (C) Chao1 and Shannon indices of bacteria and fungi. Different lowercase letters indicate significant differences between subgroups (P < 0.05). (HAR: human activity region; HIR: human inactive region).

**Table S1.** Significance test by Anosim and MRPP of soil bacterial and fungal community in different pH gradient groups.

| Groups | 16S/Anosim | | 16S/MRPP | | ITS/Anosim | | ITS/MRPP | |
| --- | --- | --- | --- | --- | --- | --- | --- | --- |
|  | R | *P* | A | *P* | R | *P* | A | *P* |
| 5.50-6.00 vs 6.00-6.50 | -0.0216 | 0.560 | -0.0013 | 0.465 | 0.0123 | 0.371 | 0.0007 | 0.335 |
| 4.00-4.50 vs 6.00-6.50 | 0.1266 | 0.115 | 0.0021 | 0.053 | 0.3083 | 0.004** | 0.0037 | 0.001** |
| 4.00-4.50 vs 5.50-6.00 | 0.0181 | 0.430 | -0.0015 | 0.958 | 0.1426 | 0.085 | 0.0013 | 0.018* |
| 3.50-4.00 vs 6.00-6.50 | 0.0878 | 0.220 | 0.0020 | 0.246 | 0.3157 | 0.004** | 0.0132 | 0.001** |
| 3.50-4.00 vs 5.50-6.00 | 0.0577 | 0.271 | 0.0005 | 0.380 | 0.1462 | 0.054 | 0.0070 | 0.002** |
| 3.50-4.00 vs 4.00-4.50 | 0.0218 | 0.332 | 0.0039 | 0.007** | 0.0990 | 0.047* | 0.0009 | 0.024* |
| 4.50-5.00 vs 6.00-6.50 | 0.0698 | 0.234 | 0.0009 | 0.130 | 0.1139 | 0.174 | 0.0016 | 0.005** |
| 4.50-5.00 vs 5.50-6.00 | -0.0120 | 0.544 | -0.0007 | 0.832 | 0.0269 | 0.362 | 0.0002 | 0.236 |
| 4.50-5.00 vs 4.00-4.50 | -0.0142 | 0.855 | 0.0005 | 0.130 | 0.0116 | 0.179 | 0.0018 | 0.001** |
| 4.50-5.00 vs 3.50-4.00 | -0.0276 | 0.687 | 0.0018 | 0.029* | 0.1129 | 0.032* | 0.0023 | 0.001** |
| 5.00-5.50 vs 6.00-6.50 | 0.1218 | 0.181 | 0.0028 | 0.119 | -0.0008 | 0.489 | 0.0013 | 0.093 |
| 5.00-5.50 vs 5.50-6.00 | 0.0517 | 0.294 | -0.0017 | 0.788 | -0.0099 | 0.513 | -0.0009 | 0.825 |
| 5.00-5.50 vs 4.00-4.50 | -0.0133 | 0.703 | 0.0001 | 0.368 | 0.1796 | 0.001** | 0.0067 | 0.001** |
| 5.00-5.50 vs 3.50-4.00 | 0.0303 | 0.244 | 0.0040 | 0.030* | 0.2058 | 0.001** | 0.0098 | 0.001** |
| 5.00-5.50 vs 4.50-5.00 | -0.0545 | 0.944 | -0.0003 | 0.649 | 0.0534 | 0.068 | 0.0022 | 0.001** |

R values between (-1, 1), and R or A values > 0 indicates that the between-groups difference is greater than the within-groups difference; R or A values < 0 indicates that the within-groups difference is greater than the between-groups difference, same below. (*, P < 0.05; **, P < 0.01, same below).

**Table S2.** Significance test by Anosim and MRPP of soil bacterial and fungal community in different altitudes groups.

| Groups | 16S/Anosim | | 16S/MRPP | | ITS/Anosim | | ITS/MRPP | |
| --- | --- | --- | --- | --- | --- | --- | --- | --- |
|  | R | *P* | A | *P* | R | *P* | A | *P* |
| 2,000 vs 2,100 | 0.0713 | 0.017* | 0.0044 | 0.033* | 0.0212 | 0.262 | 0.0035 | 0.002** |
| 2,200 vs 2,100 | 0.1956 | 0.001** | 0.0295 | 0.001** | 0.0999 | 0.001** | 0.0073 | 0.001** |
| 2,200 vs 2,000 | 0.2389 | 0.001** | 0.0244 | 0.001** | 0.1236 | 0.001** | 0.0065 | 0.001** |
| 1,300 vs 2,100 | 0.0759 | 0.190 | 0.0224 | 0.002** | 0.2890 | 0.005** | 0.0214 | 0.001** |
| 1,300 vs 2,000 | 0.2913 | 0.002** | 0.0266 | 0.001** | 0.1374 | 0.088 | 0.0111 | 0.001** |
| 1,300 vs 2,200 | 0.3051 | 0.002** | 0.0509 | 0.001** | 0.2764 | 0.008** | 0.0216 | 0.001** |
| 1,500 vs 2,100 | 0.1295 | 0.001** | 0.0165 | 0.002** | 0.2515 | 0.001** | 0.0139 | 0.001** |
| 1,500 vs 2,000 | 0.2063 | 0.001** | 0.0100 | 0.004* | 0.2088 | 0.001** | 0.0069 | 0.001** |
| 1,500 vs 2,200 | 0.1863 | 0.001** | 0.0273 | 0.001** | 0.2402 | 0.001** | 0.0119 | 0.001** |
| 1,500 vs 1,300 | 0.0112 | 0.437 | 0.0299 | 0.001** | -0.1248 | 0.889 | 0.0120 | 0.001** |
| 1,400 vs 2,100 | 0.0651 | 0.020* | 0.0168 | 0.002** | 0.3586 | 0.001** | 0.0187 | 0.001** |
| 1,400 vs 2,000 | 0.2505 | 0.001** | 0.0346 | 0.001** | 0.1945 | 0.001** | 0.0130 | 0.001** |
| 1,400 vs 2,200 | 0.3293 | 0.001** | 0.0459 | 0.001** | 0.3315 | 0.001** | 0.0154 | 0.001** |
| 1,400 vs 1,300 | -0.2152 | 0.998 | 0.0022 | 0.214 | -0.0297 | 0.596 | 0.0030 | 0.067 |
| 1,400 vs 1,500 | 0.1805 | 0.001** | 0.0243 | 0.001** | 0.2176 | 0.001** | 0.0072 | 0.001** |
| 900 vs 2,100 | 0.1056 | 0.238 | 0.0375 | 0.001** | 0.1272 | 0.182 | 0.0211 | 0.001** |
| 900 vs 2,000 | 0.2729 | 0.045* | 0.0275 | 0.001** | -0.0428 | 0.598 | 0.0109 | 0.001** |
| 900 vs 2,200 | 0.4056 | 0.003** | 0.0557 | 0.001** | 0.0249 | 0.403 | 0.0193 | 0.001** |
| 900 vs 1,300 | 0.6716 | 0.003** | 0.1225 | 0.005** | -0.1442 | 0.780 | 0.0162 | 0.097 |
| 900 vs 1,500 | 0.1184 | 0.196 | 0.0498 | 0.001** | -0.2805 | 0.974 | 0.0162 | 0.001** |

**Table S3**. Significance test by Anosim and MRPP of soil bacterial and fungal community in different regions groups.

| Groups | 16S/Anosim | | 16S/MRPP | | ITS/Anosim | | ITS/MRPP | |
| --- | --- | --- | --- | --- | --- | --- | --- | --- |
|  | R | *P* | A | *P* | R | *P* | A | *P* |
| YD vs YX | 0.0796 | 0.079 | 0.0079 | 0.001** | 0.0746 | 0.081 | 0.0046 | 0.001** |
| FQ vs YX | 0.1714 | 0.004** | 0.0099 | 0.035* | 0.0971 | 0.031* | 0.0076 | 0.003** |
| FQ vs YD | 0.2527 | 0.003** | 0.0082 | 0.003** | 0.0761 | 0.151 | 0.0035 | 0.001** |
| ZK vs YX | 0.0613 | 0.063 | 0.0167 | 0.003** | 0.1455 | 0.005** | 0.0186 | 0.001** |
| ZK vs YD | -0.1300 | 0.971 | 0.0021 | 0.079 | -0.1623 | 0.991 | 0.0050 | 0.001** |
| ZK vs FQ | 0.1872 | 0.001** | 0.0267 | 0.001** | 0.2466 | 0.001** | 0.0168 | 0.001** |
| CY vs YX | 0.2165 | 0.005** | 0.0187 | 0.012* | 0.3789 | 0.001** | 0.0095 | 0.010* |
| CY vs YD | 0.0878 | 0.249 | 0.0034 | 0.055 | 0.2736 | 0.008** | 0.0022 | 0.018* |
| CY vs FQ | -0.0227 | 0.509 | 0.0154 | 0.098 | 0.1971 | 0.017* | 0.0093 | 0.031* |
| CY vs ZK | 0.2939 | 0.010* | 0.0167 | 0.023* | 0.5623 | 0.001** | 0.0139 | 0.001** |
| GM vs YX | 0.1685 | 0.001** | 0.0228 | 0.001** | 0.1224 | 0.001** | 0.0088 | 0.001** |
| GM vs YD | 0.1170 | 0.026* | 0.0086 | 0.001** | 0.1778 | 0.004** | 0.0043 | 0.001** |
| GM vs FQ | 0.2149 | 0.004** | 0.0257 | 0.002** | 0.0464 | 0.163 | 0.0083 | 0.002** |
| GM vs ZK | 0.0083 | 0.392 | 0.0096 | 0.022* | 0.0488 | 0.150 | 0.0150 | 0.001** |
| GM vs CY | 0.1135 | 0.167 | 0.0157 | 0.025* | 0.1325 | 0.088 | 0.0052 | 0.028* |
| LX vs YX | 0.3685 | 0.001** | 0.0403 | 0.001** | 0.3286 | 0.001** | 0.0078 | 0.001** |
| LX vs YD | 0.4799 | 0.001** | 0.0744 | 0.001** | 0.2264 | 0.001** | 0.0082 | 0.001** |
| LX vs FQ | 0.5371 | 0.001** | 0.0283 | 0.001** | 0.2654 | 0.003** | 0.0038 | 0.003** |
| LX vs ZK | 0.3977 | 0.001** | 0.0432 | 0.001** | 0.1215 | 0.044* | 0.0071 | 0.001** |
| LX vs CY | 0.5015 | 0.001** | 0.0189 | 0.001** | 0.4490 | 0.001** | 0.0022 | 0.008** |
